# Case-Based Specialty Training for Medical Students to Elicit Social Determinants of Health

**DOI:** 10.15766/mep_2374-8265.11402

**Published:** 2024-05-21

**Authors:** Danielle Howell, Ellen Pearlman, Rebecca Dougherty, John Paul Sánchez, Melissa Pawelczak, Alice Fornari, Joseph S. Weiner, Robert O. Roswell

**Affiliations:** 1 First-Year Resident Physician, Department of Psychiatry, Icahn School of Medicine at Mount Sinai; 2 Associate Dean for Professionalism and Doctoring Skills, Donald and Barbara Zucker School of Medicine at Hofstra/Northwell; 3 Associate Professor, Department of Medicine, Donald and Barbara Zucker School of Medicine at Hofstra/Northwell; 4 President, Building the Next Generation of Academic Physicians (BNGAP); Executive Associate Vice President, Office for Diversity, Equity and Inclusion, Health Sciences Center, University of New Mexico; 5 Assistant Dean for the Advanced Clinical Experience, Donald and Barbara Zucker School of Medicine at Hofstra/Northwell; 6 Associate Dean for Educational Skills Development, Donald and Barbara Zucker School of Medicine at Hofstra/Northwell; 7 Clinical Associate Professor, Department of Psychiatry, Donald and Barbara Zucker School of Medicine at Hofstra/Northwell; 8 Associate Dean for Diversity, Equity, and Inclusion, Donald and Barbara Zucker School of Medicine at Hofstra/Northwell; Co-Director of Cardiology, Lenox Hill Hospital, Northwell Health

**Keywords:** Case-Based Learning, Health Equity, Internal Medicine, Neurology, OB/GYN, Pediatrics, Psychiatry, Social Determinants of Health, Surgery - General, Diversity, Equity, Inclusion

## Abstract

**Introduction:**

Without explicit education and training on how social determinants of health (SDoH) impact patient care and health outcomes, medical schools are failing to effectively equip future physicians to serve their patients. We created this workshop on health equity with a focus on SDoH to help students more effectively communicate with diverse populations.

**Methods:**

Third-year medical students and faculty were provided with class guides, learning objectives, role-play vignettes containing clerkship-specific history and physical exams, schedules, and discussion questions during a 2-hour session centered on SDoH. The workshop's impact was measured through mixed-methods analysis of surveys.

**Results:**

Based on pre- and postsurvey results from 87 participants, medical students strongly agreed that (1) SDoH factor more into a patient's health outcomes than the clinical encounter (pre: 67%, post: 87%), (2) it is their duty to gather information about SDoH (pre: 86%, post: 97%), (3) neighborhood safety is one of the key SDoH (pre: 88%, post: 97%), (4) they understood the impact of upstream interventions (pre: 35%, post: 93%), (5) they could efficiently screen all patients for SDoH at every medical encounter (pre: 27%, post: 86%), and (6) they could find preliminary resources to quickly assist patients in need of help regarding particular SDoH (pre: 26%, post: 85%).

**Discussion:**

This was the first iteration of this workshop; challenges involved piloting the content, time restraints, and organizational structure of the workshop design. Future directions include making SDoH curricula an integral part of undergraduate medical education and diverse clinical environments.

## Educational Objectives

By the end of this activity, learners will be able to:

1. Define *social determinants of health*.
2. Define *total health*.
3. Consider how the clinical team can best impact a patient's total health.
4. Construct a narrative that effectively describes a patient's social determinants of health.
5. Consider how information about a patient's social determinants of health can be utilized to improve health outcomes.

## Introduction

In recent years, research and discussion surrounding the field of social determinants of health (SDoH; more contemporaneously called social drivers of health) and its relevance to medicine and medical training have become prevalent. Although often used interchangeably, health disparities, health equity, and health justice have different implications in the context of SDoH. Health disparities can be understood as differences in health outcomes resulting from disadvantages or discrimination particular social groups have experienced compared to more advantaged groups.^1^ Addressing health equity means pursuing the elimination of these health disparities, and the goal of health equity is that every person has an equitable opportunity to achieve their full health potential, otherwise known as health justice.^1,2^

Medical schools are being called upon to address the implications of health disparities and SDoH on patient care and health outcomes.^3^ The World Health Organization Commission on Social Determinants of Health released a report calling for health care providers and government officials to improve health inequities by better addressing SDoH.^4^ The US Department of Health and Human Services (HHS) released an action plan to reduce racial and ethnic disparities in health care, which states that health disparities are closely linked with social, economic, and environmental disadvantage and that they are driven by the social conditions in which individuals live, learn, work, and play.^5^ This HHS disparities action plan provides a foundation for the Healthy People 2030 goals and objectives, as well as the National Partnership for Action stakeholder strategy, calling for a comprehensive, community-driven approach to reduce health disparities in the US and achieve health justice.^6,7^ Clinical care accounts for only 20% of an individual's health outcomes—40% can be attributed to social and economic factors like education, employment, income, family and social support, and community safety.^8^ Despite the fact that SDoH have been suggested to have a larger effect on patient care and patient outcomes than direct clinical care, in the 10 years since many of these references were published, there has been little if any literature quantifying the integration of SDoH into medical education curricula.

A commentary in *Academic Medicine* called on all medical schools to integrate the biopsychosocial model as the foundation of their programs.^9^ During the Prioritizing Health Disparities in Medical Education to Improve Care conference, medical students from across the country agreed that “the fundamental significance of making health disparities education and research a regular part of the curriculum lies in the manner in which those educational experiences are translated to patient care.”^10^ This was further supported by a statement in *Public Health Reports* that provider education on cross-cultural issues is an effective strategy to address racial and ethnic disparities in health care.^11^ Furthermore, the AAMC has supported all of these statements by emphasizing that up to this point, limited expert faculty, limited curricula, and various other factors have impeded academic medical centers from providing the education on health care disparities that is desperately needed by medical students and physicians. The AAMC states that academic health centers must commit to and be held accountable for understanding and eliminating health disparities and that reducing disparities will require broadening medical education to better train future physicians.^12^

There are sparse examples of curricular approaches that rise to the challenges presented by the above-mentioned government and academic organizations. A number of *MedEdPORTAL* publications have provided much needed curricular material on SDoH aimed at both undergraduate and graduate medical education. Specifically, Song, Poythress, Bocchini, and Kass created a mandatory curriculum on SDoH for first-year medical students during orientation.^13^ Moffett, Shahidi, Sule, and Lamba designed a three-part curriculum for a required emergency medicine student clerkship.^14^ Drake, Keeport, Chapman, and Chakraborti created a 2-year longitudinal curriculum on SDoH for a subset of first- and second-year medical students.^15^ McDonald, West, and Israel also created a curriculum on SDoH and advocacy for their second-year medical students, consisting of a combination of didactic, small-group discussion, and project work (a social work interview, a neighborhood survey, a needs and resources assessment for a patient, or an advocacy project).^16^ Lypson and colleagues described a curriculum for second-year medical students involving a case discussion of a patient with limited English proficiency, specifically focusing on gathering a social history focusing on SDoH.^17^ Most similar to our curricular initiative, Burke, Bigham, and Ferrara-Leach implemented a case-based workshop to train medical students to assess SDoH needs and connect with community resources.^18^

The Donald and Barbara Zucker School of Medicine at Hofstra/Northwell (ZSOM) engages students in a required 4-year curriculum in physician-patient communication and interpersonal skills. The curriculum is based on the three function model, articulated by Cole and Bird.^19^ This model posits that medical dialogues contain three and only three functions:

• Function one: Build the relationship.
• Function two: Assess and understand the patient's problems.
• Function three: Collaborate for management.

This model of communication is aligned with concepts of patient and family engaged care,^20^ relationship-centered care,^21^ and Engel's pioneering call for the “need for a new medical [biopsychosocial] model” of illness.^22^

What is unique about our curricular initiative compared to other curricula implemented in the past is that it involves specialty-specific cases and is required for the entire medical school class, rather than for a subset of students or students who are enrolled in an elective, have a level of preexisting knowledge of the topics discussed, or have a particular interest in the topic area. We believe this workshop is more generalizable and reproducible than other modules that have been published. It is also set apart from prior curricular initiatives because it assesses concrete skill sets of students before and after taking part in it. This measurement of acquired skills and knowledge amongst a large population of students with a high postsurvey rate is unique. Furthermore, the case-based format allows students to practice playing the role of both provider and patient and is clerkship specific. We sought to implement an OSCE format that would allow our workshop to be reproducible and feasible to implement in a wide array of medical education settings. This role-based case exercise aligns with the mission and curricular aims of the ZSOM, which was founded with the goal of departing from traditional undergraduate medical education curricula to create a focus on conceptual knowledge in action instead of memorization. Thus, the ZSOM is uniquely suited to address the call for a curricular focus on SDoH and places an emphasis on students independently developing into lifelong learners in this area. Our project-specific aim was to create a framework for teaching SDoH within core undergraduate medical education curricula that would ensure students attain skills directly transferable to clinical settings.

## Methods

### Setting/Context

The ZSOM's 4-year communication curriculum began in the first week of medical school with a core series of classes spanning 7 weeks. Andragogy contained didactics, small-group discussions, role-play, and practice dialogues with standardized patients. Subsequent topics of the communication and interpersonal skills curriculum included more advanced subjects, such as the sexual history, sharing emotionally challenging news, health information fluency, cultural humility, and communicating with children and adolescents.

Third-year medical school students had 1 week of dedicated, theme-based curriculum time, integrated on three occasions throughout the students’ clinical year. One of these themes focused on health equity and assessing how SDoH might be impacting their patients’ health. This specific curricular component is the subject of this publication.

### Session Description

The health equity-themed week for third-year medical students focused on SDoH as part of an expanded social history to inform a collaborative and effective treatment and management plan considering SDOH for the patient. The curriculum included 1 day that specifically provided students with practical skills that could be used in clinical settings to address SDoH. This session was conducted with clinical faculty from the medical school as facilitators and third-year medical students as participants but could be adapted to any clinical teaching learning environment.

### Participants

All third-year medical students and faculty involved in the third-year medical student curriculum were required to attend the session.

### Session Logistics

#### Prework

In preparation for the large- and small-group sessions, students and faculty members were provided with guides containing clerkship-specific history and physical exams, time targets specifying how the workshop should be paced, and discussion questions students and faculty should address, thus meeting the set of learning objectives defined in each handout (Appendices A and B).

Prior to the large- and small-group sessions, faculty attended a 1-hour faculty development session led by senior faculty involved in the diversity, equity, and inclusion curricular initiatives in the medical school, where they were able to review the faculty guide (Appendix A) and ask any logistical questions about the small-group session. There was no prerequisite specialized knowledge about SDoH required for facilitators or learners.

#### Session agenda

All third-year medical students and faculty first attended a large-group lecture that provided an overview of SDoH (Appendix C). After the large-group session, students gathered for a small-group session with core medical school faculty. We split the overall group of nine students into three small groups containing three students each based on the clerkship students had most recently completed (medicine/surgery, pediatrics/OB/GYN, or neurology/psychiatry). We then provided students and faculty with guides that specified a schedule, the learning objectives, and expectations (Appendices A and B).

We tailored the cases provided to the clerkship that participating students had recently completed. Each student was assigned the role of doctor, patient, or observer for the purposes of the role-play exercise. The student playing the role of doctor was asked to consider how they could best frame questions addressing SDoH in a sensitive and open-ended manner. The student playing the patient was asked to read the detailed social history provided in their student guide (Appendix B). The student playing the observer was asked to read the detailed social history and the checklist provided in the student guide (Appendix B). This checklist was used by the observer to assess their fellow students’ abilities to employ skills like expressing empathy and support, asking open-ended questions, and exploring issues of health information fluency (Appendix B).

#### Assessment

The goals of this workshop were for students to understand the importance and potential impact of SDoH on health outcomes and to develop a skills-based approach to a social history, while being mindful of the components of total health to ultimately improve health outcomes.

We used a combined qualitative and quantitative approach to critically analyze the impact of the large- and small-group portions of this session. We developed a survey (Appendices D [presurvey], E [postsurvey], and F [survey answer key]) and administered it to the students before the educational workshop (Appendix D) and after the workshop had been completed (Appendix E). We also obtained institutional review board approval (Hofstra, Approval Ref#: 20190213-SOM-ROS-1, February 13, 2019). The survey was administered through Qualtrics, an online survey program tool. We developed survey questions to assess knowledge of SDoH, attitudes towards the impact of SDoH on health outcomes, beliefs on who should be collecting SDoH data, and concerns about how collected SDoH data could be acted upon. For quantitative analysis, we formatted the options for survey responses using a 5-point Likert scale (1 = *strongly disagree,* 5 = *strongly agree*). The change in total percentage of agree or strongly agree answers in the pre- versus postsurvey was calculated. Statistical significance was calculated using the chi-square test in Microsoft Excel. To reject the null hypothesis, we deemed *p* < .05 to be statistically significant.

For qualitative analysis, we analyzed the end-of-course evaluation routinely administered to ZSOM learners. The end-of-course evaluation gave the learner the option to describe their reaction to a course. We analyzed the open-ended text in the evaluations and categorized any themes that arose about the SDoH curriculum.

## Results

We collected quantitative and qualitative data following one installment of the Health Equity Week SDoH Training. Ninety-two third-year medical students participated in the session. We administered a pre- and postsurvey at the beginning and end of the week to all participants. All 92 students completed the presurvey (100% response rate), and 87 students completed the postsurvey (95% response rate).

After the curriculum, students strongly agreed that (1) SDoH impact patients’ health outcomes more than the clinical encounter (pre: 67%, post: 87%), (2) it was part of their duty to gather information about SDoH (pre: 86%, post: 97%), (3) neighborhood safety was one of the key SDoH (pre: 88%, post: 97%), (4) they understood the impact of upstream interventions (pre: 35%, post: 93%), (5) they could efficiently screen all patients for SDoH at every medical encounter (pre: 27%, post: 86%), and (6) they could find preliminary resources to quickly assist patients in need of help from particular SDoH (pre: 26%, post: 85%; Table).

**Table.**
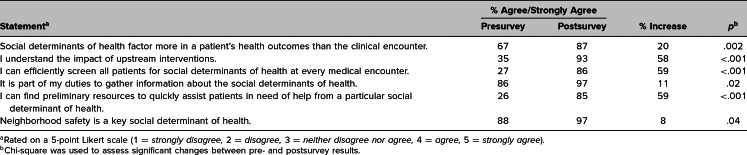
Participants’ Pre- and Postsurvey Responses (*N* = 87)

### Qualitative Themes

Students were given the option to provide written comments describing their impressions of the session. The following themes proved salient, as exemplified by the corresponding quotes:

• Gratitude that SDoH were included in the curriculum:
  ○ “I felt very grateful that Hofstra added this critical topic to our curriculum. I left this session feeling enlightened and empowered.”
• The clinical relevance of learning about SDoH:
  ○ “The case-based small-group session felt realistic and very practical, and the history taking was much more straightforward than I expected, yielding a lot of clinically relevant information that I could act on.”
• The need for further curricular initiatives addressing SDoH:
  ○ “At the end of the session I still did feel kind of helpless in terms of addressing these different social determinants of health.”
  ○ “Felt like a lot of social work responsibilities—unclear of the balance between med student and social work.”

## Discussion

Our case-based session on SDoH was successfully implemented as a required part of the third-year medical student curriculum at the ZSOM. There has been increased demand for curricula that directly address SDoH so that future physicians are equipped with tools to better serve their patients. However, despite the evidence that biopsychosocial forces greatly affect a patient's health, medical education curricula continue to favor drawing on a biomedical framework, without the important interplay between the root of a patient's social risk for, response to, and experience with a disease and the science behind a given pathology. We believe that without explicit education about social context and SDoH, medical schools are failing to effectively equip their future physicians to serve their patients, and our workshop aims to address this deficiency.

We accomplished our aim and demonstrated that integration of SDoH into the ZSOM curriculum enabled students to recognize SDoH impacts so that they could potentially provide more equitable care to their patients with cultural humility. From the qualitative analysis, we learned there is a need for further curricular initiatives addressing SDoH and more effectively defining the roles health care providers can take to act on the social needs uncovered in patients through SDoH screenings. Furthermore, we learned that future development of these curricular initiatives is needed to better empower students to address social needs identified in clinical settings and to promote a culture where addressing SDoH is considered the responsibility of all health care providers.

From the analyses, the strengths of this session include its seamless integration with the larger curricular frameworks of the medical school with respect to encouraging patient-centered interactions. This curricular initiative is unique in that it has been integrated into a 4-year curriculum and adapted into an OSCE format that provides easy adaptation and reproducibility in other school curricula. Furthermore, our session stands out in that it includes cases catering to the clerkship in which each particular student has most recently participated. One of the challenges of implementing this session was that this was the first iteration, which thus involved piloting the content, time restraints, and organizational structure of the workshop design. We recommend providing the teaching materials to faculty members prior to the session and holding space for them to ask questions about the workshop prior to implementation to ensure the session runs smoothly.

The limitations of this curricular initiative include having completed only one iteration of the workshop and thus not having longitudinal data to support its efficacy. We would have love to explore topics like insurance or health care access in our interviews, but we were unfortunately limited by the time constraints of our module and were able to include only a portion of the five broad areas of SDoH in our interview checklist. Our data are also limited because they are based on self-reported knowledge, without an objective basis to measure the skill set gained through participation. Thus, it is difficult to conclude whether this skill set will have a lasting impact on the way these students approach clinical care in the future. Future implications of our work include making SDoH curricula an integral part of undergraduate medical education and implementing this format of teaching SDoH in other medical school settings. Given the qualitative student feedback, we also recommend adapting this session to feature interprofessional role-play. Including other members of the health care team, such as social workers and care coordinators, has the potential to prevent feelings of helplessness and clearly delineate the way team members with differing roles in hospital settings can work together to address SDoH.

In summary, through pre- and postsurvey results, our curricular initiative has demonstrated self-reported efficacy in equipping medical students with the tools to provide more equitable care with cultural humility. The increases from pre- to postsurvey results demonstrate that, while the students did not feel strongly about the learning objectives before the session, after the session they felt they understood the importance of SDoH, could screen for them, and were proficient in finding resources to assist patients in need of a resource pertaining to SDoH. Though translation to the bedside and clinic was not measured, the confidence students felt about entering the workforce with knowledge of the importance of SDoH and assessing social needs in their respective patient populations yields optimism that integrating this kind of initiative into undergraduate medical education can have a substantial effect on the kind of providers students become after graduation.

## Appendices


Faculty Guide.docxStudent Guide.docxIntro to SDoH.pptxPresurvey.docxPostsurvey.docxSurvey Answer Key.docx
*All appendices are peer reviewed as integral parts of the Original Publication.*
