# Supplementary material for: Case-Based Specialty Training for Medical Students to Elicit Social Determinants of Health
Source: MedEdPORTAL. 2024 May 21;20:11402. doi: 10.15766/mep_2374-8265.11402 (PMC11219088; doi:10.15766/mep_2374-8265.11402)
Supplement: Supplementary file 1 — Faculty Guide.docxStudent Guide.docxIntro to SDoH.pptxPresurvey.docxPostsurvey.docxSurvey Answer Key.docx [file mep_2374-8265.11402-s001.zip › B. Student Guide.docx]

**Social Determinants of Health-History**

**Taking Appendix for Students**

Note that this Appendix *is solely for your reference* and contains all of the information that the students are provided for each of the 3 cases (Medicine pages 2-7, Neuro pages 8-12, Pediatrics pages 13-18): All students will receive:

1. The student guide and HPI (note that these

questions are similar for all 3 cases)

The student playing the patient will additionally

receive:

1. The historical information

The student playing the observer will additionally receive:

1. The historical information

2. The checklist

1

**Guide for Medicine/Surgery Students**

***Part 1: Case Discussion***

Read the attached case and discuss the following 2 questions:

1. What are the possible reasons for this patient’s recurrent admissions?

2. Which social determinants of health may be contributing to this patient’s health outcomes?

***Part 2: Role Play***

You will now practice taking a history around social determinants of health. While these cases are fictitious, they are based on real-life scenarios that may resonate with participants of this workshop. Please be mindful as you approach this activity and remain actively engaged in this role play as this is an opportunity to strengthen your ability to address clinical scenarios you will encounter in your future training. Choose among yourselves which role each of you will take and perform the following prior to starting the role play:

1. Doctor-Consider how you will best frame your questions around social determinants of health in a sensitive and open ended manner.

2. Patient-Read the detailed social history provided.

3. Observer-Read the detailed social history provided and the checklist. Use the checklist during the role play and take notes.

***Part 3: Identify next steps and early interventions and Prepare case presentation***

1. Discuss the next 2 questions:

a. The patient is concerned about being evicted from her home. She lives in the 11435 zip code. Use the housing services tab in the following web resource to find eviction prevention services 5-10 miles from the patient’s home: www.hitesite.org (or use any site specific to your state that provides a resource directory for free and low-cost health and social services)

b. What steps could have been taken earlier in this patient’s care to prevent the downstream consequences to this patient’s health?

2. Prepare a brief (under 7 minute) patient presentation for the whole group and designate one person to give the presentation. Your presentation should include:

a. A brief medical summary

b. The social determinants of health that impacted this patient’s clinical outcomes c. Preventative measures that could have been taken to improve this patient’s health d. Next steps for this patient using the information gained from the website including a discharge plan

2

***Part 4: Putting it All Together***

Our goal is to improve the total health of our patients. Research shows that: Overall or total health = 20% Clinical Care +30% Health Behaviors (Diet/Sexual Activity, Exercise) + 40% Socioeconomic factors (Employment, Income, Community Safety) + 10% Physical Environmental Factors (Air quality, Housing)

• We know that time constraints make it impossible to cover all aspects of patients’ social determinants of health. Given all that we have covered today, are there one or two questions that could open the conversation with patients around social determinants and allow you to hone in on those that are most relevant?

**Medicine Case**

**HPI**

Ms Ford is a 56 y/o woman with hypertension, hyperlipidemia who presents with a 2 days history of worsening shortness of breath and fatigue. Patient states that she was in her usual state of health until one week ago when she noted more dyspnea than usual. The symptoms have worsened over the past 2 days so she presented to the emergency department for evaluation.

Review of Systems – neg except for above

Past Medical History/Past Surgical History – as above

MEDS – Valsartan 80 mg daily; Amlodipine 10mg daily

No Known Drug Allergies

Social History - ½ pk/day cigarette h/o, no Etoh, no illicit drugs

Family History- non-contributory

Exam

BP 145/82 Heart Rate85 Respiratory Rate 14 98% on Room Air

Gen: No acute distress

Neck: No Jugular Venous Distension

CV: Heart Rate regular rate and rhythm, no murmurs, rubs, or gallops Lungs: Clear to auscultation bilaterally

Abd: Soft, nontender, nondistended

Ext: No clubbing, cyanosis, or edema

3

LABS/STUDIES

CBC: unremarkable

BMP: 141/4.1 100/26 21/0.9

D Dimer: Negative

Lipase: Negative

Hgb A1c: 6.1%

LDL 140 HDL 38

Troponin I: positive

EKG: Normal Sinus Rhythm; Normal axis and intervals; diffuse 2 mm ST depressions, ST elevations AVR

Transthoracic Echocardiogram: Ejection Fraction40%

Chest X-Ray– bibasilar pulmonary edema

Cath: Triple vessel disease

Recommendation: CABG Surgery

Day 3: Pt undergoes successful CABG

Day 8: Pt d/c’d home with f/u in 2 weeks

*D/C Meds*:

Aspirin, Clopidogrel, Sacubitril-Valsartan, Atorvastatin, Furosemide , Wound dressing changes

Day 11, Readmission #1: Pt readmitted for decompensated heart failure three days post discharge.

Discharge: Emphasized that Furosemide is to be taken twice daily. Emphasized to continue the new medication Sacubitril-Valsartan as it just hit the market and is proven to decrease recurrent decompensated congestive heart failure (CHF)admissions & mortality.

Day 18, Readmission #2: Pt readmitted for decompensated CHF and sternal wound infection with possible wound dehiscence one week following discharge.

Antibiotics started, diuretics and afterload reduction started.

4

**Medicine Case**

**Answers to Historical Questions**

**You are the patient**

***Housing Issues***

You are concerned about your health and are struggling to make ends meet. When you were first discharged from the hospital, you were given a medication and didn’t quite understand that it would make you use the bathroom often. You are living in an apartment that currently requires significant plumbing work. The toilet is functional at times but is in the process of being repaired. The plumbing work leaves the apartment in a very unsanitary condition. Due to the work, the landlord is in the process of trying to evict you to charge higher rent for your unit.

***Diet Issues***

None. You work some shifts at a local diner and usually have salads with tuna or turkey. You adhere to a low sodium diet.

***Medication Issues***

Furosemide

⮚ caused you to urinate quite frequently

⮚ Because of the plumbing issues, you decided to stop taking this medication regularly ⮚ You took the medication early in the morning getting ready to one of your jobs, and had an episode of bladder incontinence. That was the final straw! You have discontinued taking that medication altogether.

Sacubitril-Valsartan

⮚ Wasn’t given to you as it had to be approved through your insurance and needed prior authorization.

Wound Dressing

⮚ The wound dressing changes also weren’t covered (medical supplies)

⮚ the pharmacist instructed you to purchase it at the pharmacy, it’s just $19.99 or $34.99. ⮚ stretched thin for cash, figured you would use the few gauzes that you had and change them less often.

**Readmission #1**

You quickly started to realize that you were getting dyspneic again. You were unsure about next steps and ultimately had to re-present to the hospital for evaluation and treatment. You were given lasix and realized the importance of a working toilet. You were grateful to receive dressing

5

changes in the hospital as you noticed that your sternal wound was starting to redden and discolor. You figured that with these dressing changes, your wound would get better.

You were ready to be discharged home and to continue fighting this eviction. **Discharge #2**

Sacubitril-Valsartan

⮚ not covered by your insurance and would cost $725/month for you.

⮚ Medication not an option for you at this time

Wound Dressing

⮚ stretch out the usage of the wound dressings.

To your delight, the bathroom was fixed! You had decided to take the Lasix pills when you were home near the toilet once daily. You are looking for more stable employment and are constantly on the go.

Your apartment has the new toilet, but it is more chaotic since you’ve not been there because of the readmission to the hospital. You are growing more and more concerned as eviction notices are piling up and the landlord is not answering calls at this point.

You begin feeling fatigued again, but also feverish. Your shortness of breath has recurred and the wound from the surgery is now inflamed and pustulent. You have one last gauze and you cleaned off the debris and put the gauze on the sternal wound. The next couple of days, the dyspnea worsens and the wound looks like it’s starting to separate with the expression of pus. You are very seriously concerned about being evicted from your home, but ultimately present to the ER again.

**Medicine Case Checklist**

**Checklist for Observer-please circle yes or no and write comments for each question**

1. Did the doctor express empathy to the patient? (Consider reflection, legitimation, and exploration) **Yes/No**

2. Did the doctor express partnership and/or support? **Yes/No**

3. Did the doctor use open-ended questions? **Yes/No**

6

4. Did the doctor explore the patient’s living situation including housing security and its impact on Ms. Ford’s health? (Where does the patient live and what is her daily routine? Is the patient domiciled or at risk for homelessness?) **Yes/No**

5. Did the doctor explore the family’s economic situation and its impact on Ms. Ford’s health? (Can the patient afford the entresto, are there economic barriers to obtaining medication and medical supplies?) **Yes/No**

6. Did the doctor explore Ms. Ford’s diet? (How are meals prepared? How often does she eat? Is she following a low sodium diet?) **Yes/No**

7. Did the doctor explore issues of health information fluency? (Does the patient understand her medical condition? Does she understand the prescribed medications including expected effects, most common side effects and how to take them?) **Yes/No**

7

**Guide for Neuro/Psych Students**

***Section 1: Case Discussion***

Read the attached case and discuss the following questions:

1. What are the possible reasons for this patient’s admission?

2. Which social determinants of health may be contributing to this patient’s health outcomes?

***Section 2: Role Play***

You will now practice taking a history around social determinants of health. Choose among yourselves which role each of you will take and perform the following prior to starting the role play:

1. Doctor- Consider how you will best frame your questions around social determinants of health in a sensitive and open ended manner.

2. Patient-Read the detailed social history provided.

3. Observer-Read the detailed social history provided and the checklist. Use the checklist during the role play and take notes.

***Section 3: Identify next steps, early interventions, and prepare case***

***presentation***

1. Discuss the next 2 questions:

a. The family needs coordination of care and services. They live in the 11501 zip code. Use the Social Support and Services to Care Coordination Services tabs in the following web resource to find eviction prevention services 1-5 miles from the patient’s home: www.hitesite.org (or use any site specific to your state that provides a resource directory for free and low-cost health and social services)

b. What steps could have been taken earlier in this patient’s care to prevent the downstream consequences to this patient’s health?

2. Prepare a brief (under 7 minute) patient presentation for the group and designate one person to give the presentation. Your presentation should include:

a. A brief medical summary

b. The social determinants of health that impacted this patient’s clinical outcomes g. Preventative measures that could have been taken to improve this patient’s health h. Next steps for this patient using the information gained from the website including a discharge plan

8

***Part 4: Putting it All Together***

Our goal is to improve the total health of our patients. Research shows that: Overall or total health = 20% Clinical Care +30% Health Behaviors (Diet/Sexual Activity, Exercise) + 40% Socioeconomic factors (Employment, Income, Community Safety) + 10% Physical Environmental Factors (Air quality, Housing)

• We know that time constraints make it impossible to cover all aspects of patients’ social determinants of health. Given all that we have covered today, are there one or two questions that could open the conversation with patients around social determinants and allow you to hone in on those that are most relevant?

**Neurology Case**

**HPI**

Walter Lewis is a 77 year old man with a past medical history significant for diabetes, hypertension, hyperlipidemia and osteoarthritis who presents with right sided weakness and slurred speech to the emergency room. The patient is accompanied by his wife who reports that on waking she noticed her husband was struggling to get out of bed and that his speech was garbled.

**Review of Systems:** notable for some increased urinary frequency per his wife, otherwise negative

**Past Medical History/Past Surgical History** – as noted above

**MEDS** – metformin 1000 mg bid, Norvasc 10mg, simvastatin 20 mg daily and ibuprofen as need for joint pain

**Allergies: Sulfa**

**Social History** – 25 pack year tobacco history, quit at 45, daily alcohol use, 2 scotch drinks

**Family History**– non-contributory

**Exam**

• VS: afebrile, Blood Pressure 240/158, Heart Rate88, Respiratory Rate 20, Pulse Ox98% on Room Air

• Gen: No acute distress

• Awake, Alert, oriented to person only

• CV: Exam notable for 3/6 systolic ejection murmur, regular rate and rhythm, otherwise within normal limits

• Lungs: Clear to auscultation bilaterally

9

• Abd: soft nontender, nondistended

• Neuro: Right upper extremity Strength 4/5, right lower extremity strength 3/5, dysarthria noted

EKG: no acute ST segment changes

Labs:

• Wbc 7.0, hgb 11, plt 320, Na 140, K 3.2, Cr 1.2, glucose 245

Imaging:

• MRI: LeftMCA infarct

**Hospital Course**

• During his hospital course the patient is started on aspirin and his statin dose is increased. His blood pressure is controlled and he undergoes an echo which is unremarkable. On hospital day 2, the patient is noted to be febrile and a CXR reveals a right lower lobePneumonia. He undergoes a speech and swallow evaluation and thickened liquids and a pureed diet are recommended as are aspiration precautions. The patient is seen by PT and inpatient rehab is recommended.

**Rehab Stay**

• During his rehab stay the patient progresses, completing two weeks of therapy and is discharged to home with a rolling walker. During rehab the patient is noted to have some erythema on his sacrum.

**Readmission**

• The patient is readmitted one week following discharge having fallen when attempting to climb the stairs from the 1st floor of his home to the second floor. Additionally, he is found to have skin breakdown on his buttocks which is now painful. He also notes a cough and has a temperature of 100.5.

**Neurology Case**

**Answers to Historical Questions**

**You are the patient**

***Economic Concerns***

- You and your wife have been supporting your son’s family as your son recently lost his job. You are using your pension to help cover your son’s mortgage payments. You are concerned because you think you may need to private hire some aids to help out at home

10

with shopping and some grooming but are not sure you will be able to pay for these services given your son’s current situation.

***Housing Concerns***

• You are concerned about your ability to navigate your current home. There are five steps from your walkway to the front door and your bedroom is on the second floor. You have been struggling to get up the stairs to your bedroom and have spent many nights since discharge from the rehabilitation center sleeping on the couch.

***Diet Issues***

• You typically took care of the grocery shopping. Your wife has severe rheumatoid arthritis and is quite frail. You would take the car to the store once a week, but since the stroke you are no longer able to drive and aren’t as confident with the public transportation lines. Your neighbors have been helping out with shopping, but you don’t think you can depend on them long term. You did not receive any training regarding how to prepare a pureed diet and have been eating mostly yogurt and soup.

***Medication Issues***

• At the rehabilitation center you were told that the area just above your sacrum was red. You have not seen the area since discharge as it has been difficult for you to bathe and you have been taking sponge baths in the kitchen. You think you were told to put a cream on the area, but aren’t sure what it was.

***Community and Social Context:***

• You wear glasses and are hard of hearing although you have not had a formal hearing evaluation.

• You have long term relationships with neighbors, but they are all facing similar health concerns and both of your children live out of state.

**Readmission #1**

• You realize that the diet you were eating was not what they are serving you in the hospital and you notice that the nurses have paid a lot of attention to the red area above your sacrum. You feel weaker than you did when you left the rehab center but worry about spending more time away from home and from your wife. You are frustrated that your strength has not improved.

11

**Neurology Case Checklist**

**Checklist for Observer-please circle yes or no and write comments for each question**

1. Did the doctor express empathy to the patient? (Consider reflection, legitimation, and exploration) **Yes/No**

2. Did the doctor express partnership and/or support? **Yes/No**

3. Did the doctor use open-ended questions? **Yes/No**

4. Did the doctor explore Mr. Lewis’s living situation and its impact on his health? (Consider where the patient is sleeping, how the patient is able to get to the grocery store given that he can no longer drive, food preparation, his role as care-giver to his wife) **Yes/No**

5. Did the doctor explore Mr. Lewis’s economic situation and its impact on his health? (Consider food choice, ability to pay for home health aids, financial support patient is providing to his son and his son’s family) **Yes/No**

6. Did the doctor explore the family’s neighborhood environment and its impact on Mr. Lewis’s health? (Consider accessibility of public transportation, community resources) **Yes/No**

7. Did the doctor explore issues of health information fluency? (Consider Mr. Lewis’s understanding of his pureed diet, or wound care recommendations) **Yes/No**

12

**Guide for Pediatrics/OBGYN Students**

***Section 1: Case Discussion***

Read the attached case and discuss the following questions:

1. What is the differential diagnosis for this patient’s problem?

2. Which social determinants of health may be contributing to this patient’s health outcomes?

***Section 2: Role Play***

You will now practice taking a history around social determinants of health. Choose among yourselves which role each of you will take and read the following prior to starting the role play:

1. Doctor- Consider how you will best frame your questions around social determinants of health in a sensitive and open ended manner.

2. Patient-Read the detailed social history provided.

3. Observer-Read the detailed social history provided and the checklist. Use the checklist during the role play and take notes.

***Section 3: Identify next steps and early interventions and Prepare case presentation***

1. Discuss the next 2 questions:

a. The family needs to find recreational resources for Bobby that are safe. They live in the 11549 zip code. Use the youth and services tab in the following web resource to find services. Look for 10 miles from the patient’s home: www.hitesite.org (or use any site specific to your state that provides a resource directory for free and low-cost health and social services)

b. What steps could have been taken earlier in this patient’s care to prevent the downstream consequences to this patient’s health?

2. Prepare a brief (under 7 minute) patient presentation for the group and designate one person to give the presentation. Your presentation should include:

a. A brief medical summary

b. The social determinants of health that impacted this patient’s clinical outcomes c. Preventative measures that could have been taken to improve this patient’s health d. Next steps for this patient using the information gained from the website

13

***Part 4: Putting it All Together***

Our goal is to improve the total health of our patients. Research shows that: Overall or total health = 20% Clinical Care +30% Health Behaviors (Diet/Sexual Activity, Exercise) + 40% Socioeconomic factors (Employment, Income, Community Safety) + 10% Physical Environmental Factors (Air quality, Housing)

• We know that time constraints make it impossible to cover all aspects of patients’ social determinants of health. Given all that we have covered today, are there one or two questions that could open the conversation with patients around social determinants and allow you to hone in on those that are most relevant?

**Pediatrics Case**

**The history was obtained from the patient’s mother. The patient is doing his homework in the waiting room.**

**HPI**

Bobby Vasquez is an 8 year old boy seen in an outpatient peds clinic because his teacher said he recently seems very tired and is occasionally falling asleep in school. Mom says that she has not noticed a difference in his behavior or energy level at home but that she is often at work. He sleeps from 9:30pm to 6:15am on school days, no recent change. On weekends he falls asleep later and wakes around 11am. He recently failed a math test which is unusual for him. No recent illnesses.

**Review of Systems:** neg except for snoring every night for the past 2 years

**Birth History**: born full term, normal spontaneous vaginal delivery, no complications, birth weight 8 pounds 2 ounces

**Developmental History**: Met all milestones on time

**Immunizations:** Up to date except no flu shot

**Past Medical History/Past Surgical History** – reactive airway disease as an infant and toddler, has not required meds since age 3 years, no surgeries

**MEDS** – none

**No Known Drug Allergies**

14

**Social History** – 3^rd^ grade, gets extra help for reading, lives with Mom, brother, grandmother, aunt and maternal cousin, no smokers in house, 6+ hours of screen time per day

**Dietary History:**

► Breakfast: no breakfast, drinks water

► Lunch: school lunch (pizza, nachos, bagel with cream cheese), chocolate or strawberry milk

► After School Snack: chocolate chip cookies or Cheese Its, iced tea

► Dinner: lots of rice, fried eggs, plantains, beans, salad (sometimes), water, iced tea, the family has pizza and soda once per week

► Before bed: Either more of what he ate for dinner or Doritos

**Family History**– Mother had gestational diabetes and currently has hyperlipidemia and hypertension, 21 year old brother with hypertension and obesity, 12 year old brother with obesity, father’s history unknown

**Exam**

Height 135 cm 75%, Weight 43 kg 99%, BMI 23.6 99^th^%

Blood Pressure 100/60 Heart Rate80 both normal for age

Gen: interactive, in no acute distress

HEENT: Tympanic Membrane-normal light reflex bilaterally, 2+ tonsils with no exudate

Neck: no lymphadenopathy, thyroid not palpable

CV: heart rate regular, no murmurs, rubs, or gallops

Lungs: clear to auscultation bilaterally

Abd: soft nontender, nondistended, no hepatosplenomegaly

GU: Tanner 1 pubic hair and testes

Ext: no cyanosis, clubbing, or edema

Skin: no lesions

**Growth Charts:** since age 3 years, growth charts show a consistent height percentile with increasing weight percentile and BMI consistently above the 95% and rising

15

**Pediatrics Case**

**Answers to Historical Questions**

**You are Bobby’s mother, you are very tired and in a rush and stressed because you need to get out of the clinic soon to get to work. Bobby is in the waiting room doing his homework.**

You have not noticed that Bobby is more tired than usual at home but you work a lot. You brought him in today because the teacher called the school nurse who said you had to bring him in to the pediatrician because he’s been falling asleep in school and recently seems very tired.

**School**

► Bobby is in the 3^rd^ grade at the local elementary school. He does “okay in school.” He failed his math test last week which was unusual for him. You’re not sure why-he studies on his own because you are at work

**Activity Level**

► Screen time: 6+ hours per day of video games, plus 30 minutes of homework ► Bus to school

► Little to no physical exercise outside of gym class for 45 minutes 2-3 times per week. You want Bobby to exercise and not spend so much time on his tablet but you don’t feel like you can change it-this is very stressful. Despite this, you know Bobby is overweight but he doesn’t look that different from his friends or the rest of the family-you don’t consider his weight too unhealthy. You’re not sure if his doctor has mentioned it in the past.

► He loves to play soccer but there is no school team and the town team costs too much to join

► You live in a one family home with a backyard and there is a park close by but you feel that the neighborhood is too unsafe to allow him to go outside and play. There were multiple recent armed robberies in the neighborhood and you aren’t taking any chances.

► **Diet:** You buy what’s cheap and what the family knows how to cook. His grandmother or 15 year old cousin does most of the cooking as you are frequently at work.

► Breakfast: no breakfast (he wakes up late because he is tired and is in a rush-no time to eat before his cousin rushes him on to the bus), drinks water

► Lunch: free school lunch (pizza, nachos, bagel with cream cheese), chocolate or strawberry milk

16

► After School Snack: chocolate chip cookies or Cheese Its, iced tea

► Dinner: lots of rice, fried eggs, plantains, beans, salad (if you can afford it that week), water, iced tea, the family has pizza and soda once per week

► Before bed: Either more of what he ate for dinner or Doritos

► **Home life**

► Family is from Honduras, you moved to the US when Bobby was 1 year old because there was a lot of violence back home

► Family lives in Hempstead

► Parents are separated, he sees his father a few times a year but dad lives out of state. Father provides no financial support.

► Lives with you, 12 year old brother, maternal aunt, 15 year old female cousin, and grandmother on the top floor of a single family, rented house. You share a kitchen with another family that lives downstairs.

► No smokers

► When he comes home from school, he is with his grandmother or, most often, his 15 year old cousin who is often preoccupied with her own school work and social life.

► No smokers in house

► You work 2 jobs as a home health aid and taxi driver- even with working this much you are still struggling to make ends meet. This is very stressful. You aren’t receiving or aware of any services that could help you. Your hours are:

► Monday-Wednesday 8am-8pm

► Friday and Sat 6pm-6am

► **Sleep** Bobby shares a room with his older brother and you. He usually plays on his tablet as he is falling asleep. Bobby goes to bed at around 9:45 pm, wakes up at 6am for school, no recent changes. His brother goes to sleep around 11pm. You get sleep whenever you can-no consistent hours. Bobby wakes up a lot when you go to bed even though you try to be quiet.

**Pediatrics Case Checklist**

**Checklist for Observer-please circle yes or no and write comments for each question**

1. Did the doctor express empathy to the mother? (Consider reflection, legitimation, and exploration) **Yes/No**

17

2. Did the doctor express partnership and/or support? **Yes/No**

3. Did the doctor use open-ended questions? **Yes/No**

4. Did the doctor explore the family’s living situation and its impact on Bobby’s health? (Consider sleeping environment, food preparation, and support for homework) **Yes/No**

5. Did the doctor explore the family’s economic situation and its impact on Bobby’s health? (Consider food choice, options for activities, availability for homework support) **Yes/No**

6. Did the doctor explore the family’s neighborhood environment and its impact on Bobby’s health? (Consider safety, parks, community resources) **Yes/No**

7. Did the doctor explore issues of health information fluency? (Consider mother’s understanding of Bobby’s understanding of health issues) **Yes/No**

18
