# Supplementary material for: Case-Based Specialty Training for Medical Students to Elicit Social Determinants of Health
Source: MedEdPORTAL. 2024 May 21;20:11402. doi: 10.15766/mep_2374-8265.11402 (PMC11219088; doi:10.15766/mep_2374-8265.11402)
Supplement: Supplementary file 1 — Faculty Guide.docxStudent Guide.docxIntro to SDoH.pptxPresurvey.docxPostsurvey.docxSurvey Answer Key.docx [file mep_2374-8265.11402-s001.zip › C. Intro to SDoH.pptx]

## Slide 1
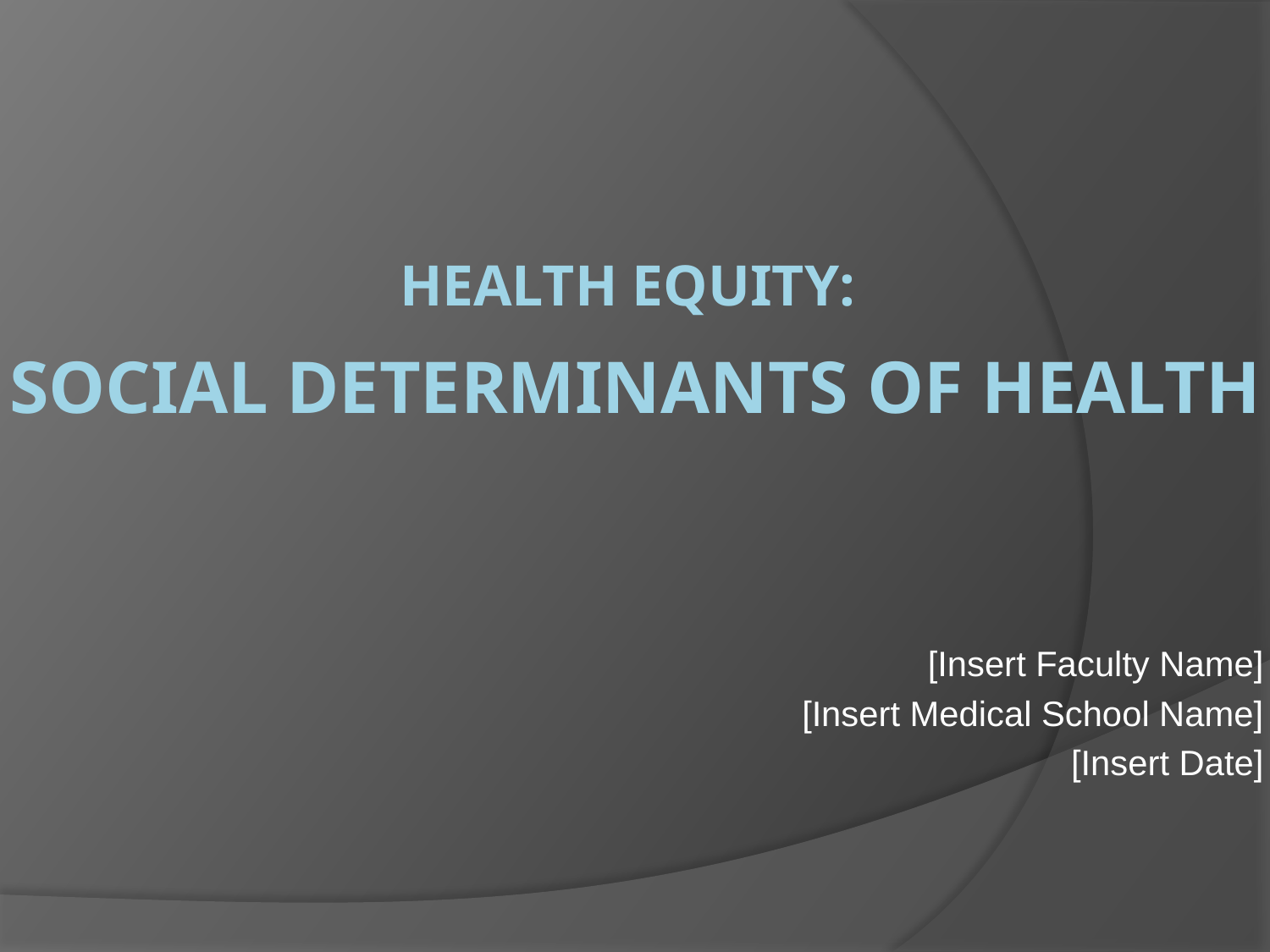

# HEALTH EQUITY: SOCIAL DETERMINANTS OF HEALTH
[Insert Faculty Name]
[Insert Medical School Name]
[Insert Date]

## Slide 2
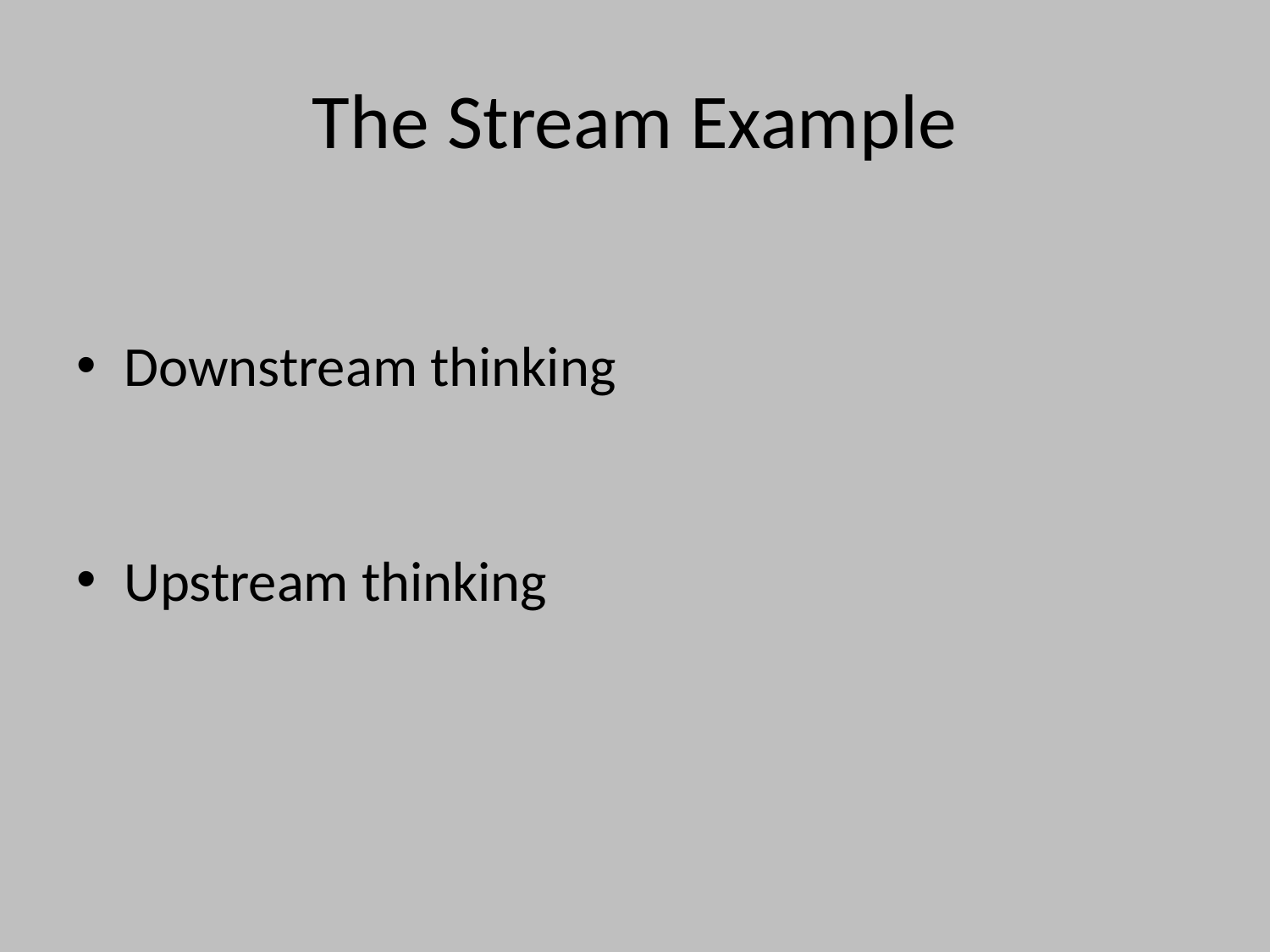

# The Stream Example
Downstream thinking
Upstream thinking

## Slide 3
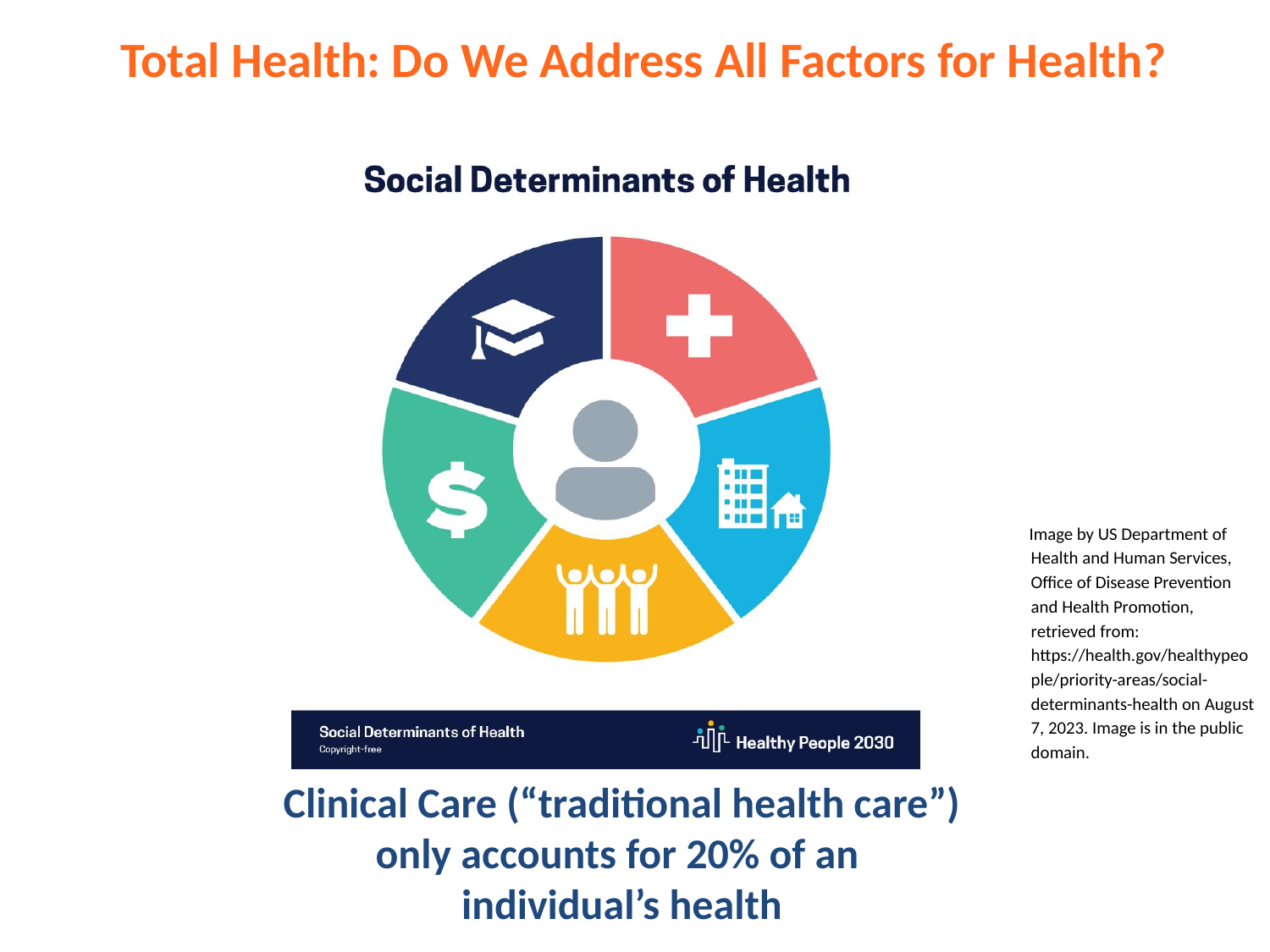

Total Health: Do We Address All Factors for Health?
Image by US Department of Health and Human Services, Office of Disease Prevention and Health Promotion, retrieved from: https://health.gov/healthypeople/priority-areas/social-determinants-health on August 7, 2023. Image is in the public domain.
Clinical Care (“traditional health care”) only accounts for 20% of an
individual’s health

## Slide 4
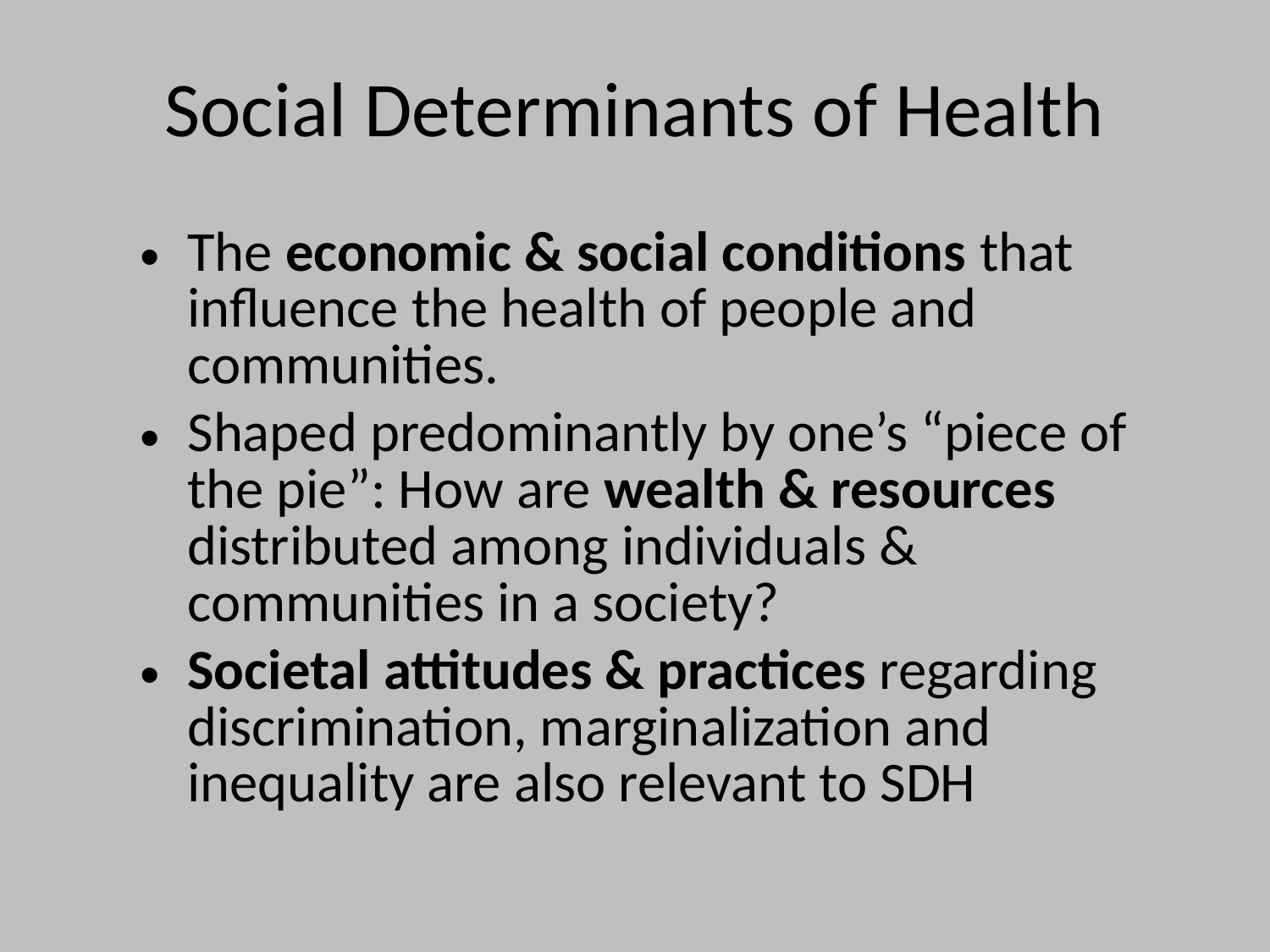

# Social Determinants of Health
The economic & social conditions that influence the health of people and communities.
Shaped predominantly by one’s “piece of the pie”: How are wealth & resources distributed among individuals & communities in a society?
Societal attitudes & practices regarding discrimination, marginalization and inequality are also relevant to SDH

## Slide 5
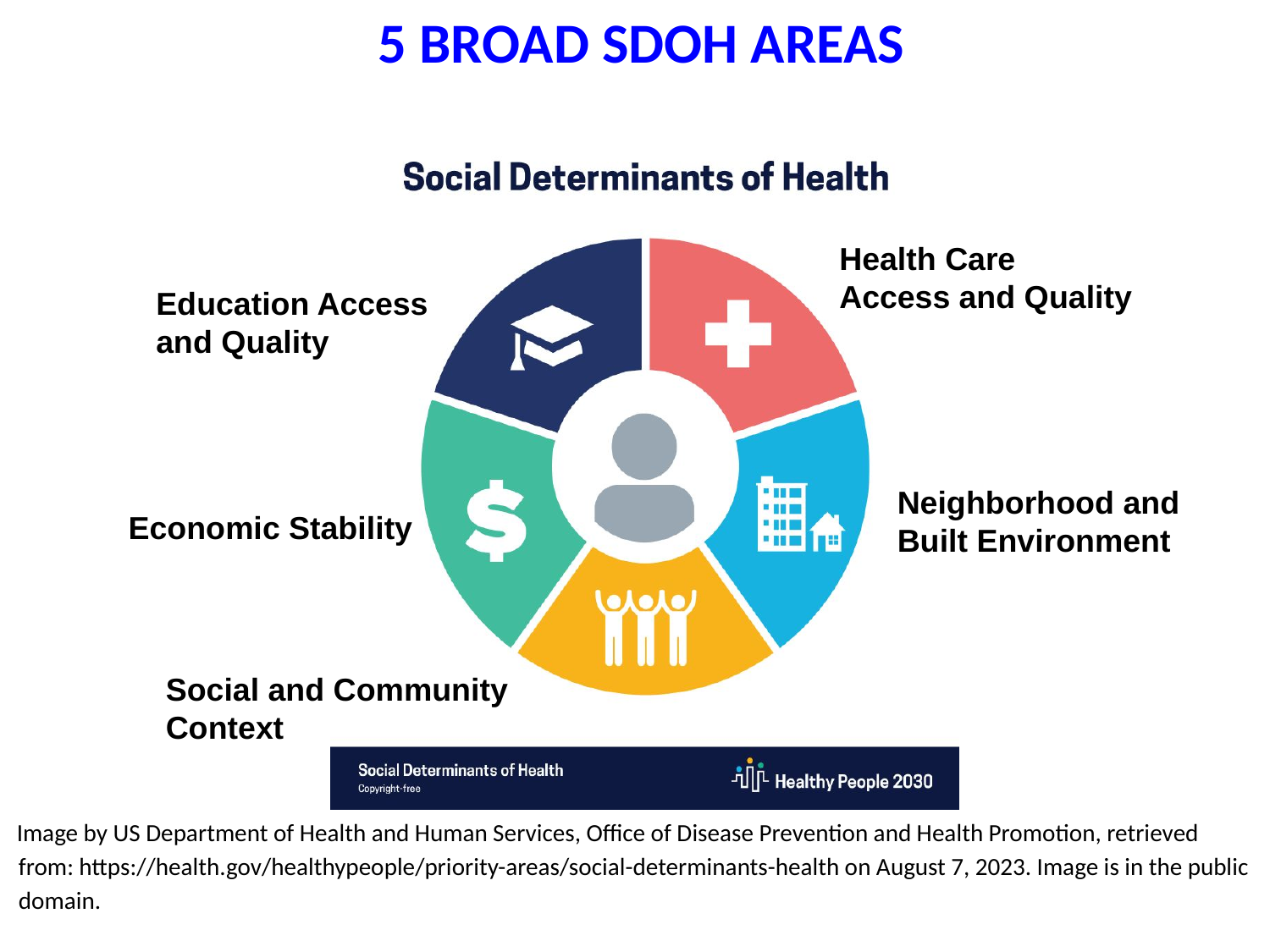

5 BROAD SDOH AREAS
Health Care Access and Quality
Education Access and Quality
Neighborhood and Built Environment
Economic Stability
Social and Community Context
Image by US Department of Health and Human Services, Office of Disease Prevention and Health Promotion, retrieved from: https://health.gov/healthypeople/priority-areas/social-determinants-health on August 7, 2023. Image is in the public domain.

## Slide 6
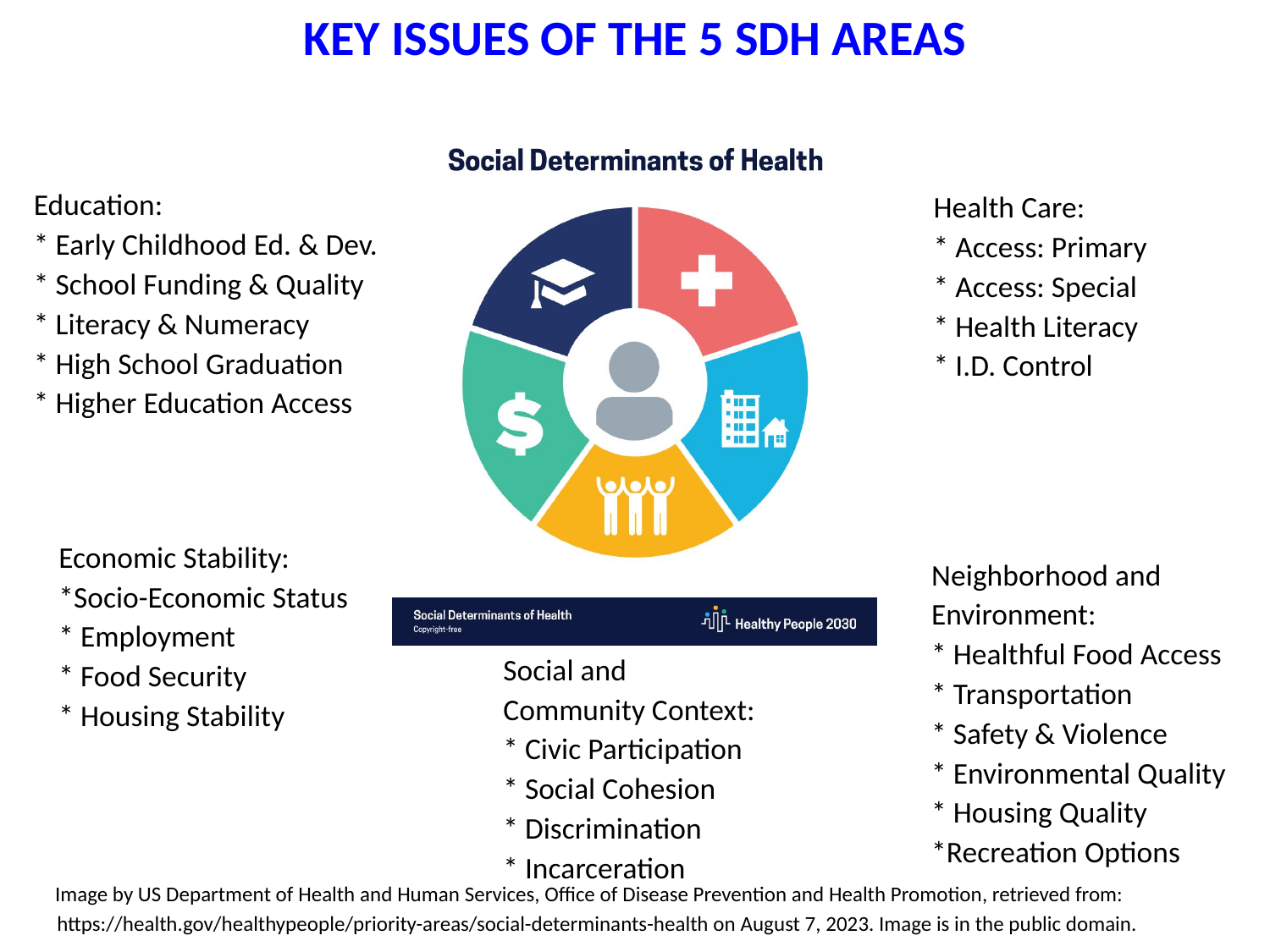

KEY ISSUES OF THE 5 SDH AREAS
Education:
* Early Childhood Ed. & Dev.
* School Funding & Quality
* Literacy & Numeracy
* High School Graduation
* Higher Education Access
Health Care:
* Access: Primary
* Access: Special
* Health Literacy
* I.D. Control
Economic Stability:
*Socio-Economic Status
* Employment
* Food Security
* Housing Stability
Neighborhood and Environment:
* Healthful Food Access
* Transportation
* Safety & Violence
* Environmental Quality
* Housing Quality
*Recreation Options
Social and Community Context:
* Civic Participation
* Social Cohesion
* Discrimination
* Incarceration
Image by US Department of Health and Human Services, Office of Disease Prevention and Health Promotion, retrieved from: https://health.gov/healthypeople/priority-areas/social-determinants-health on August 7, 2023. Image is in the public domain.

## Slide 7
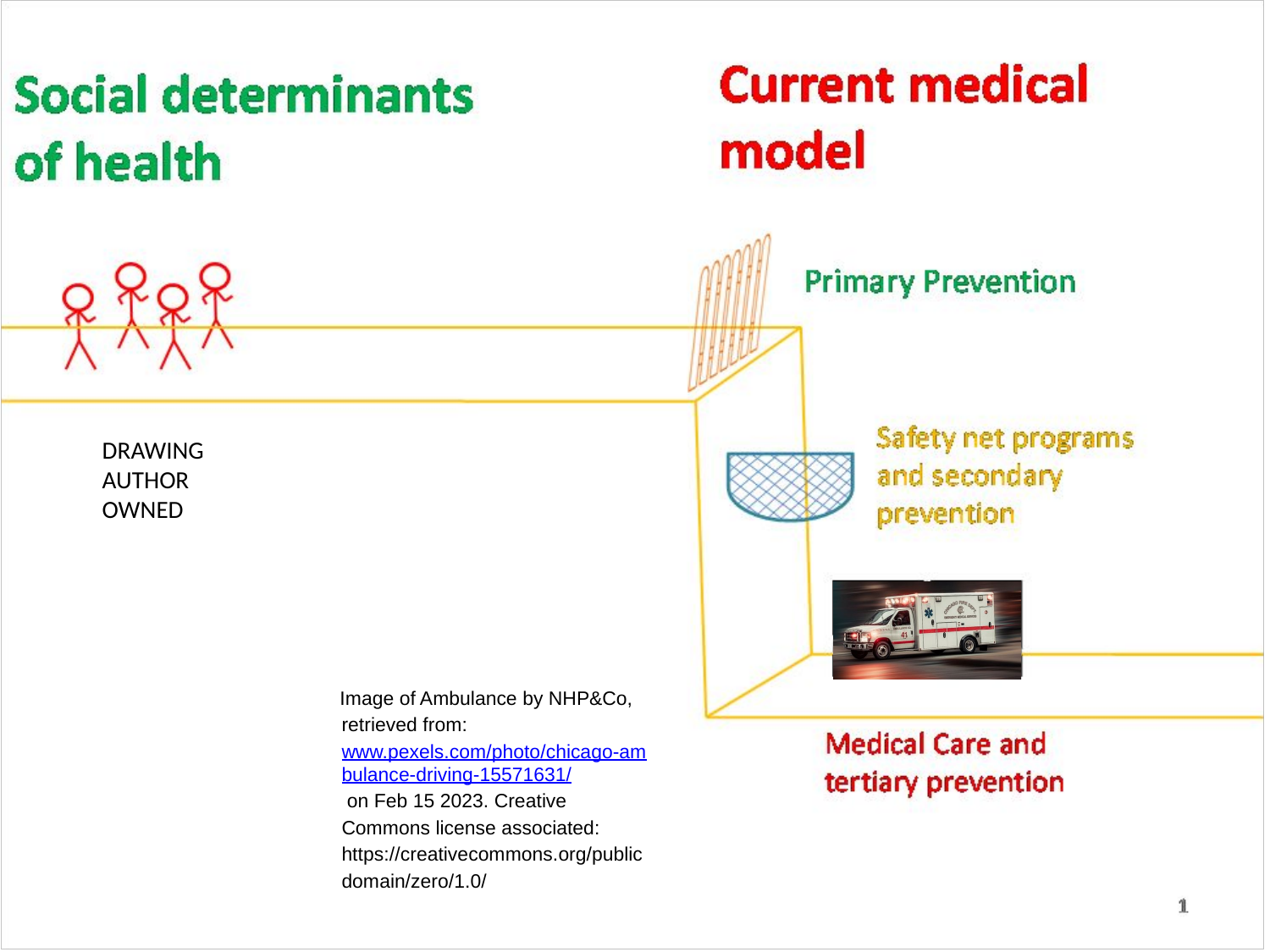

DRAWING AUTHOR OWNED
Image of Ambulance by NHP&Co, retrieved from: www.pexels.com/photo/chicago-ambulance-driving-15571631/ on Feb 15 2023. Creative Commons license associated: https://creativecommons.org/publicdomain/zero/1.0/

## Slide 8
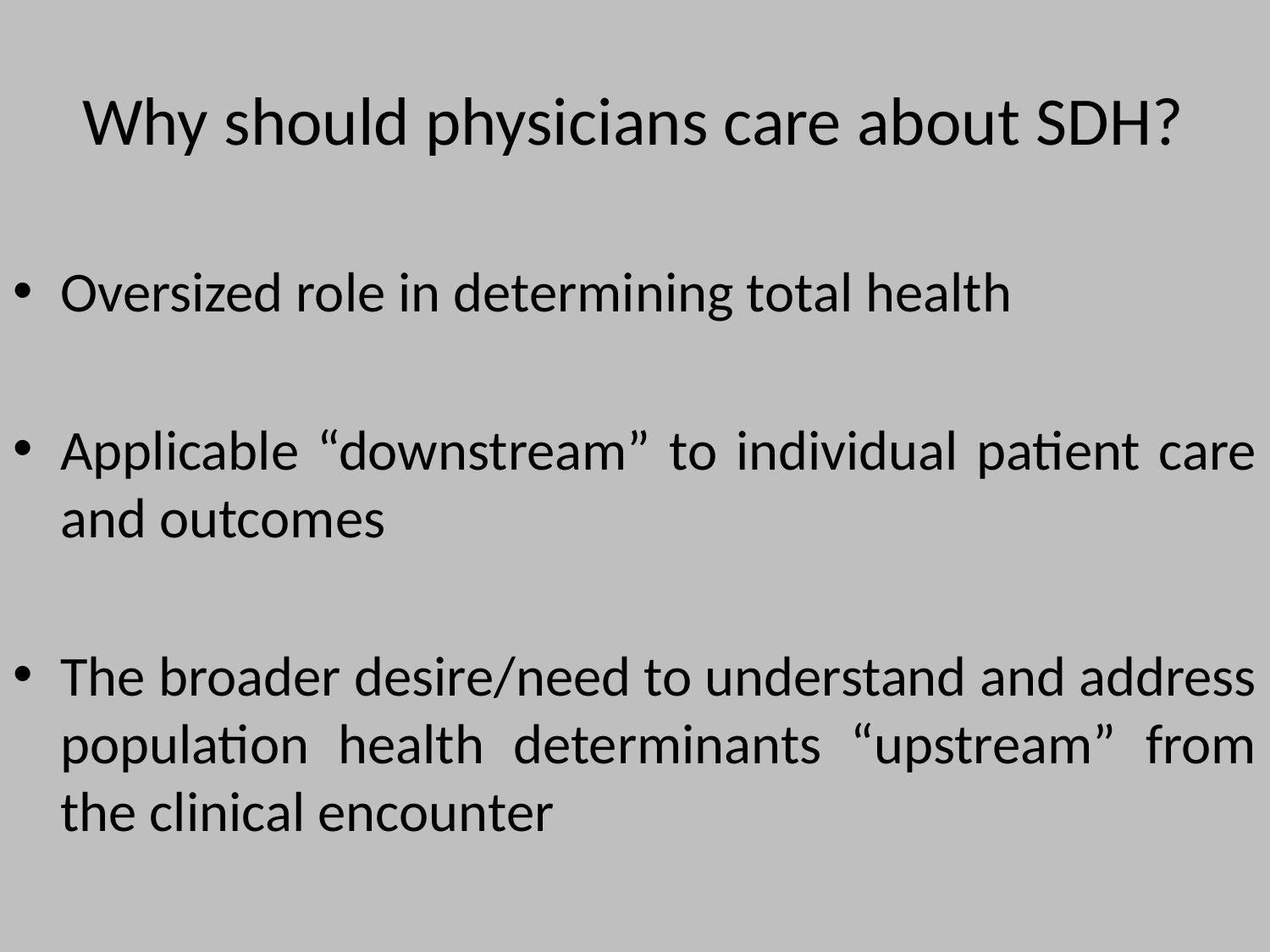

# Why should physicians care about SDH?
Oversized role in determining total health
Applicable “downstream” to individual patient care and outcomes
The broader desire/need to understand and address population health determinants “upstream” from the clinical encounter

## Slide 9
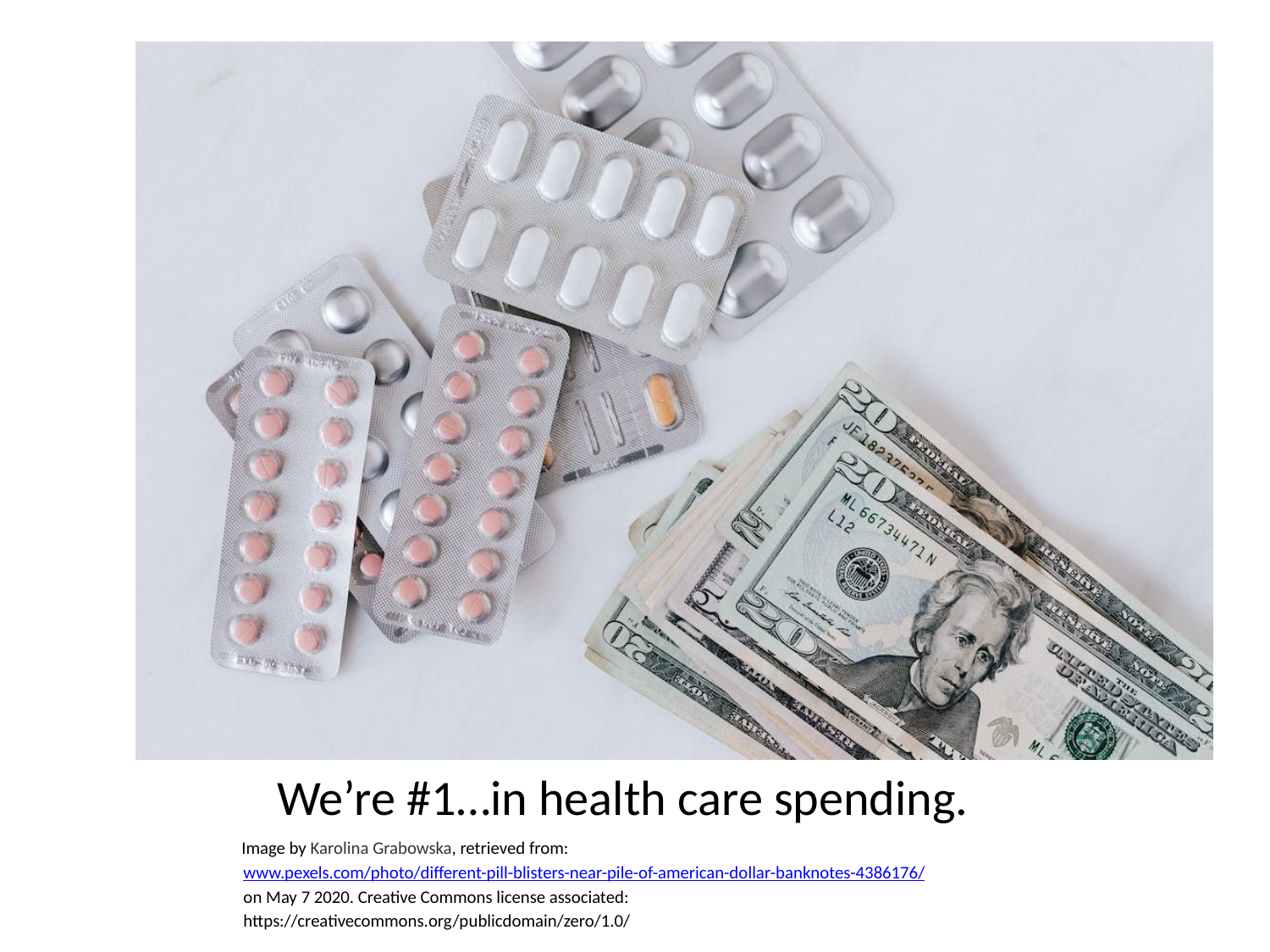

We’re #1…in health care spending.
Image by Karolina Grabowska, retrieved from: www.pexels.com/photo/different-pill-blisters-near-pile-of-american-dollar-banknotes-4386176/ on May 7 2020. Creative Commons license associated: https://creativecommons.org/publicdomain/zero/1.0/

## Slide 10
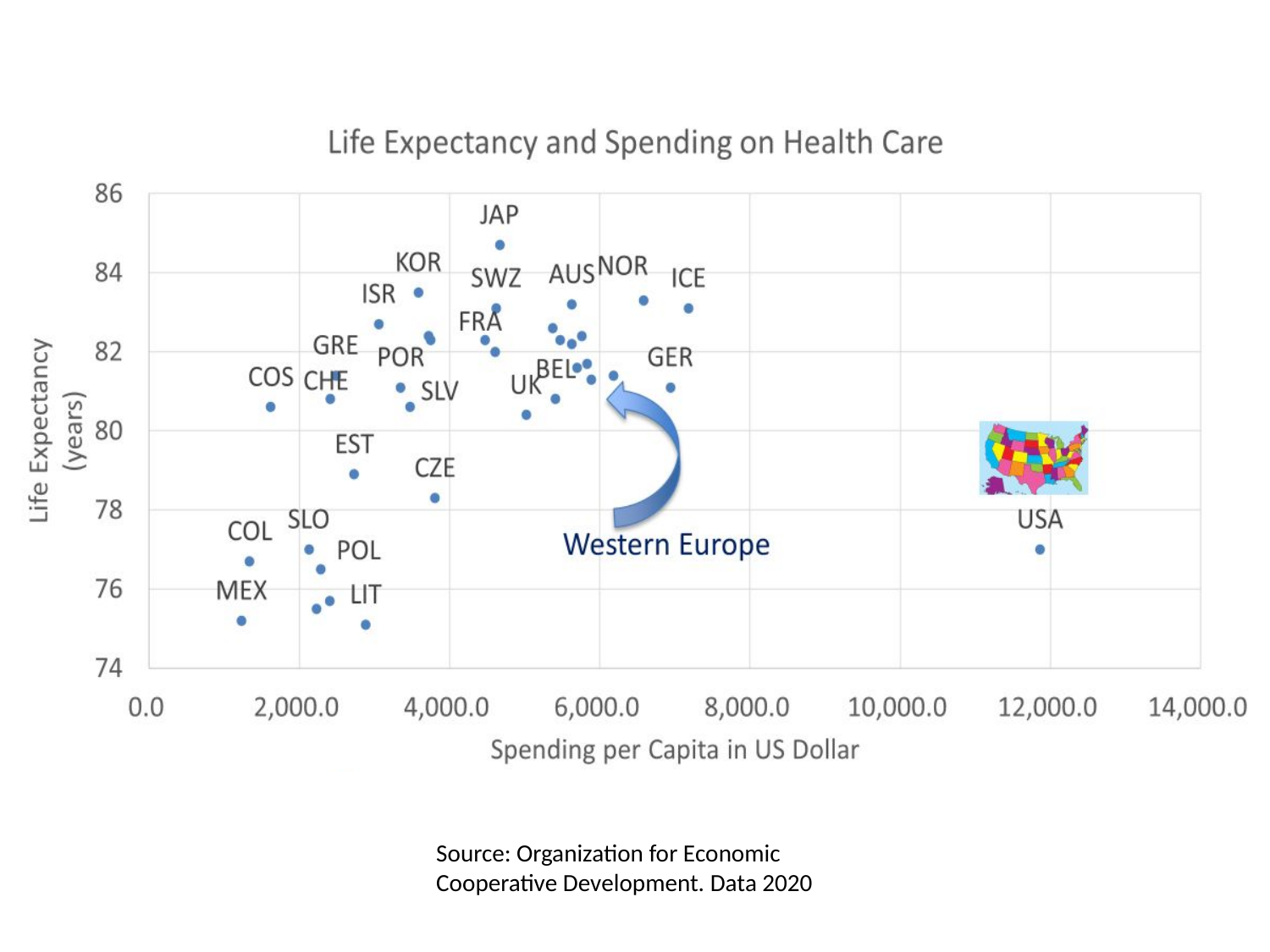

Source: Organization for Economic Cooperative Development. Data 2020

## Slide 11
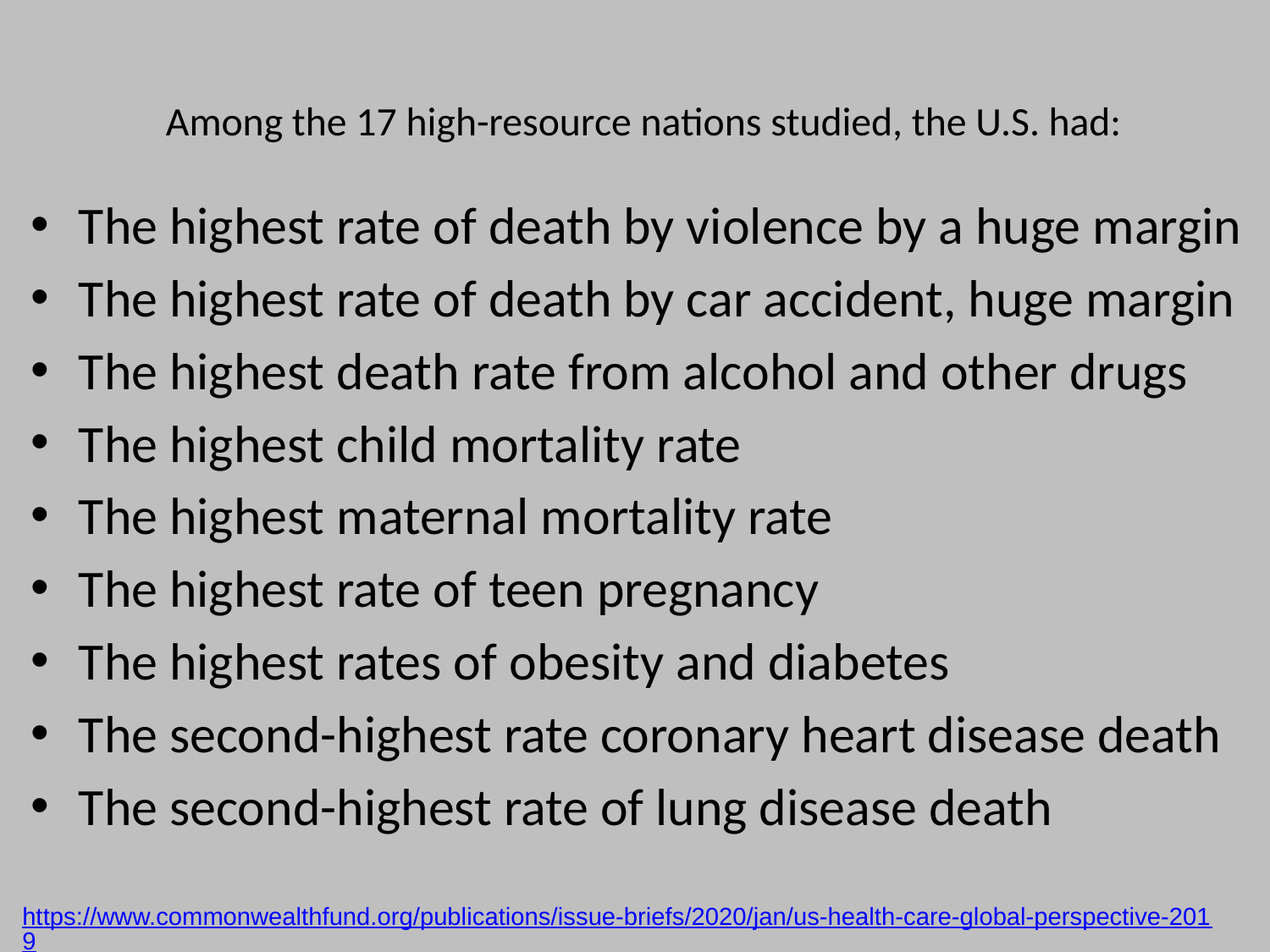

# Among the 17 high-resource nations studied, the U.S. had:
The highest rate of death by violence by a huge margin
The highest rate of death by car accident, huge margin
The highest death rate from alcohol and other drugs
The highest child mortality rate
The highest maternal mortality rate
The highest rate of teen pregnancy
The highest rates of obesity and diabetes
The second-highest rate coronary heart disease death
The second-highest rate of lung disease death
https://www.commonwealthfund.org/publications/issue-briefs/2020/jan/us-health-care-global-perspective-2019

## Slide 12
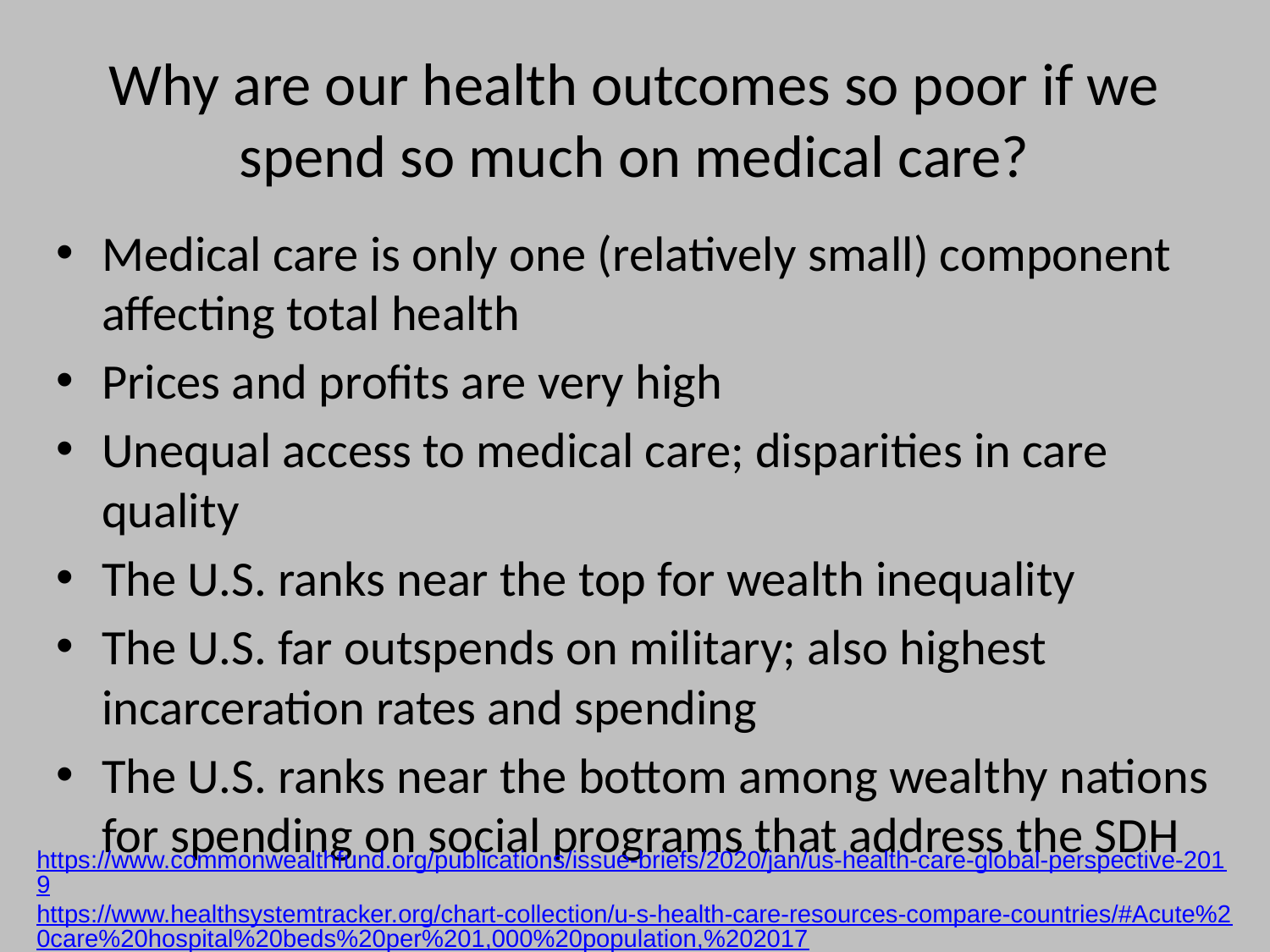

# Why are our health outcomes so poor if we spend so much on medical care?
Medical care is only one (relatively small) component affecting total health
Prices and profits are very high
Unequal access to medical care; disparities in care quality
The U.S. ranks near the top for wealth inequality
The U.S. far outspends on military; also highest incarceration rates and spending
The U.S. ranks near the bottom among wealthy nations for spending on social programs that address the SDH
https://www.commonwealthfund.org/publications/issue-briefs/2020/jan/us-health-care-global-perspective-2019
https://www.healthsystemtracker.org/chart-collection/u-s-health-care-resources-compare-countries/#Acute%20care%20hospital%20beds%20per%201,000%20population,%202017

## Slide 13
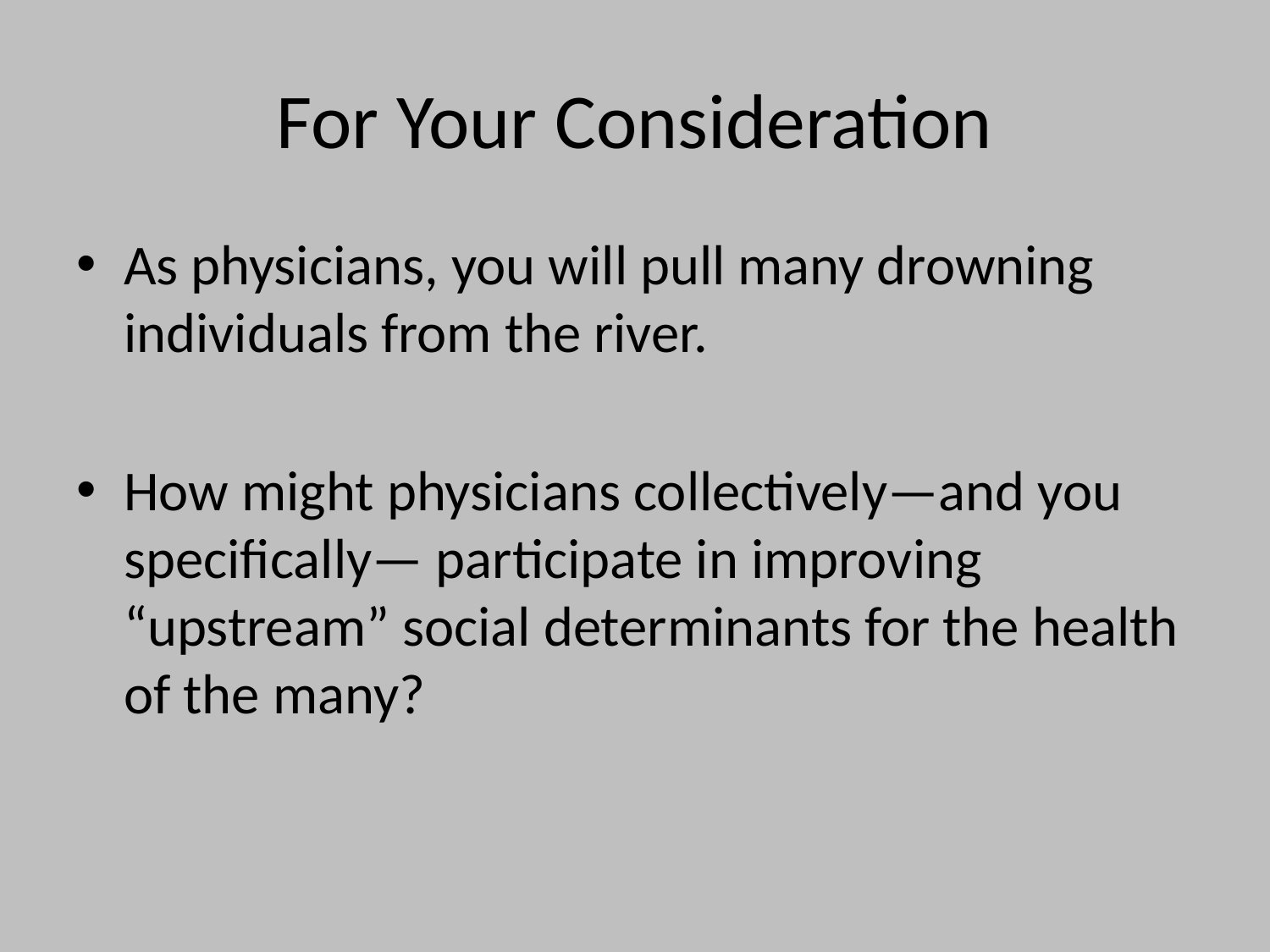

# For Your Consideration
As physicians, you will pull many drowning individuals from the river.
How might physicians collectively—and you specifically— participate in improving “upstream” social determinants for the health of the many?
