# Supplementary material for: Case-Based Specialty Training for Medical Students to Elicit Social Determinants of Health
Source: MedEdPORTAL. 2024 May 21;20:11402. doi: 10.15766/mep_2374-8265.11402 (PMC11219088; doi:10.15766/mep_2374-8265.11402)
Supplement: Supplementary file 1 — Faculty Guide.docxStudent Guide.docxIntro to SDoH.pptxPresurvey.docxPostsurvey.docxSurvey Answer Key.docx [file mep_2374-8265.11402-s001.zip › E. Postsurvey.docx]

Health Equity Themed Week Post-Survey

**RECORD THE NUMBER FROM THE PAPER ON YOUR DESK. TAKE A PICTURE ON YOUR PHONE OF YOUR SURVEY NUMBER FOR THE POST-SURVEY AT THE END OF THE SESSION.**

**This ensures all surveys are anonymously recorded.**

**Survey Number**

Section 1

1. **I am an:**

- MS3 (1)
- Other (2)

1. **What sex were you assigned at birth?**

- Male (1)
- Female (2)
- Other (please specify) (3): ______

1. **What best describes your current gender identity?**

- Man (1)
- Woman (2)
- Trans man (3)
- Trans woman (4)
- Agender (5)
- Genderqueer/Gender non-conforming (6)
- Non-binary (7)
- Another gender not listed (please specify) (8): _____

1. **I self identify as:**

- Hispanic, Latino, or of Spanish Origin (1)
- American Indian or Alaska Native (2)
- Asian (3)
- Black or African American (4)
- Middle Eastern or North African (5)
- Native Hawaiian or Other Pacific Islander (6)
- White (7)
- Other (please specify) (8): _____

Section 2: Please rate whether you strongly disagree, disagree, neither disagree nor agree, agree, or strongly agree with the provided statements below.

| Strongly Disagree (1) | | Disagree (2) | Neither Disagree nor Agree (3) | Agree (4) | Strongly Agree (5) |
| --- | --- | --- | --- | --- | --- |
| Health Equity is defined as achieving equal health outcomes | o | o | o | o | o |

| Strongly Disagree (1) | | Disagree (2) | Neither agree nor disagree (3) | Agree (4) | Strongly agree (5) |
| --- | --- | --- | --- | --- | --- |
| US health care initiatives have dramatically improved efficiency in medical spending for predicted mortality rates | o | o | o | o | o |

| Strongly Disagree (1) | | Disagree (2) | Neither agree nor disagree (3) | Agree (4) | Strongly agree (5) |
| --- | --- | --- | --- | --- | --- |
| Governance & policies are key components of achieving equity | o | o | o | o | o |



| Strongly Disagree (1) | | Disagree (2) | Neither agree nor disagree (3) | Agree (4) | Strongly agree (5) |
| --- | --- | --- | --- | --- | --- |
| The US ranks around the mean in terms of health care outcomes compared to other developed countries | o | o | o | o | o |

| Strongly Disagree (1) | | Disagree (2) | Neither agree nor disagree (3) | Agree (4) | Strongly agree (5) |
| --- | --- | --- | --- | --- | --- |
| The US doesn’t spend enough money on health care | o | o | o | o | o |



| Strongly Disagree (1) | | Disagree (2) | Neither agree nor disagree (3) | Agree (4) | Strongly agree (5) |
| --- | --- | --- | --- | --- | --- |
| Social determinants of health factor more in a patient’s health outcomes than the clinical encounter | o | o | o | o | o |

| Strongly Disagree (1) | | Disagree (2) | Neither agree nor disagree (3) | Agree (4) | Strongly agree (5) |
| --- | --- | --- | --- | --- | --- |
| I understand the impact of upstream interventions | o | o | o | o | o |

| Strongly Disagree (1) | | Disagree (2) | Neither agree nor disagree (3) | Agree (4) | Strongly agree (5) |
| --- | --- | --- | --- | --- | --- |
| I can efficiently screen all patients for social determinants of health at every medical encounter | o | o | o | o | o |



| Strongly Disagree (1) | | Disagree (2) | Neither agree nor disagree (3) | Agree (4) | Strongly agree (5) |
| --- | --- | --- | --- | --- | --- |
| Decreasing the amount of people without medical insurance will solve most, if not all of the issues with healthcare | o | o | o | o | o |

| Strongly Disagree (1) | | Disagree (2) | Neither agree nor disagree (3) | Agree (4) | Strongly agree (5) |
| --- | --- | --- | --- | --- | --- |
| It is part of my duties to gather information about the social determinants of health | o | o | o | o | o |

| Strongly Disagree (1) | | Disagree (2) | Neither agree nor disagree (3) | Agree (4) | Strongly agree (5) |
| --- | --- | --- | --- | --- | --- |
| I can find preliminary resources to quickly assist patients in need of help from a particular social determinant of health | o | o | o | o | o |

| Strongly Disagree (1) | | Disagree (2) | Neither agree nor disagree (3) | Agree (4) | Strongly agree (5) |
| --- | --- | --- | --- | --- | --- |
| I am familiar with how I can lobby for change in healthcare policy | o | o | o | o | o |



| Strongly Disagree (1) | | Disagree (2) | Neither agree nor disagree (3) | Agree (4) | Strongly agree (5) |
| --- | --- | --- | --- | --- | --- |
| Neighborhood safety is a key social determinant of health | o | o | o | o | o |

Section 3: Please answer each short answer question to the best of your ability.

**What is an upstream intervention?**

**What is total health?**
