# Supplementary material for: Case-Based Specialty Training for Medical Students to Elicit Social Determinants of Health
Source: MedEdPORTAL. 2024 May 21;20:11402. doi: 10.15766/mep_2374-8265.11402 (PMC11219088; doi:10.15766/mep_2374-8265.11402)
Supplement: Supplementary file 1 — Faculty Guide.docxStudent Guide.docxIntro to SDoH.pptxPresurvey.docxPostsurvey.docxSurvey Answer Key.docx [file mep_2374-8265.11402-s001.zip › F. Survey Answer Key.docx]

**Health Equity Themed Week Pre and Post Survey: Answer Key**

- **Section 1: Demographic Information**
  - For questions 1-4, please ask students to answer to the best of their ability in accordance with their comfort level. There are no right or wrong answers for this section.
- **Section 2: Likert scale questions**
  - In this section, options for survey responses are formatted into a 5-point Likert scale that range from Strongly Disagree to Strongly Agree. There are no right or wrong answers for this section as they assess the opinions and comfort level of participants with respect to the subject matter.
- **Section 3: Short Answer Responses**
  - These questions are included in both the pre and post survey as a measure of skills and knowledge gained through the workshop. As such, we recommend against providing official answers until after the conclusion of the workshop.
  - Ideal Answers:
    - What is an upstream intervention?
      - Answer: Upstream interventions can be defined as initiatives that seek to address the underlying causative factors that lead to poor health, typically through action on broader structural, political, economic and environmental determinants
    - What is total health?
      - Answer: Total health can be defined as encompassing the physical and social contributors to the wellbeing of individuals, families, and communities, including but not limited to socioeconomic, environmental, and behavioral health.
  - Scoring Rubric

|  | 2-Point Response | 1-Point Response | 0-Point Response |
| --- | --- | --- | --- |
| Q1: What is an upstream intervention? | Includes valid inferences or claims based on the information provided in the workshop.  Fully and directly describes the definition of an upstream intervention. | Includes inferences or claims that are loosely based on the information provided in the workshop  Responds partially to the prompt or does not address all elements of the definition of an upstream intervention. | Does not address any of the aspects of the definition of an upstream intervention or is totally inaccurate. |
| Q2: What is total health? | Includes valid inferences or claims based on the information provided in the workshop.  Fully and directly describes the definition of total health. | Includes inferences or claims that are loosely based on the information provided in the workshop  Responds partially to the prompt or does not address all elements of the definition of total health. | Does not address any of the aspects of the definition of total health or is totally inaccurate. |
