# Supplementary material for: Case-Based Specialty Training for Medical Students to Elicit Social Determinants of Health
Source: MedEdPORTAL. 2024 May 21;20:11402. doi: 10.15766/mep_2374-8265.11402 (PMC11219088; doi:10.15766/mep_2374-8265.11402)
Supplement: Supplementary file 1 — Faculty Guide.docxStudent Guide.docxIntro to SDoH.pptxPresurvey.docxPostsurvey.docxSurvey Answer Key.docx [file mep_2374-8265.11402-s001.zip › A. Faculty Guide.docx]

**Social Determinants of Health-History**

**Taking Appendix for Faculty**

**Title of Session: Social Determinants of Health-History Taking**

**[Insert Date and Time]**

***This appendix should be provided to facilitators prior to the start of this session to provide them with the goals, learning objectives, session agenda, and guidelines for this workshop. The student guide should accordingly be provided to the student participants at the start of the session.***

**Goal:**

To understand the importance and potential impact of social determinants of health on health outcomes.

To develop an approach to taking a social history while being mindful of the components of total health to ultimately improve health outcomes.

**Learning Objectives:**

Define social determinants of health.

Define total health.

Consider how the clinical team can best impact patients’ total health.

Construct a narrative that effectively describes the patient’s social determinants of health.

Consider how information about patients’ social determinants of health can be utilized to improve health outcomes.

**Prework:**

No required pre-reading for students

Faculty prework: Read through this guide and review the HITE (Health Information Tool for Empowerment) site resources as denoted in Section 3.

**Session Agenda:**

• Large group, [Insert Time]

o [Insert name of faculty member who will be teaching large group]

• Small groups, [Insert Time]

**Detailed Session Agenda:**

**Large Group: (15 minutes)**

[Insert faculty] will:

• Discuss US Health Care Costs and Health Outcomes

• Define social determinants of health

• Define upstream/downstream interventions

• Provide reasons why taking a history surrounding the social determinants is important

• Define total health

**Transition to Small Groups (5 minutes)**

**Small group: (1 hour 40 minutes)**

*Role of Faculty Member:*

• To frame sections

• To explain student roles

• To keep time and keep session on track

• To make teaching points and ensure all points are covered

• **To take attendance-it is mandatory for students to attend**

*Group Structure:*

• Groups will be comprised of approximately 3 students entering Medicine/Surgery in the next trimester, 3 students entering Pediatrics/ObGyn and 3 students entering Neurology/Psychiatry.

• Each group of students will be discussing and role playing a case pertinent to one of the 2 fields they are entering in their next trimester. For example, the group entering Medicine/Surgery will be discussing and role playing an internal medicine case. All 3 cases will be presented to the group toward the end of the session.

• While the specialty-specific cases included in our curriculum are intended to strengthen participants’ appreciation of SDoH in varying clinical contexts, these cases can be useful regardless of the specialty participants are familiar with and can be used as a general tool to frame discussions around social determinants of health.

***Section 1: Case Discussion (15 minutes)***

• Tell students to split into triads based upon the clerkship they are entering (some groups may have 2 or 4 students):

o One Med/Surg group

o One Peds/OB group

o One Neuro/Psych group

• Handout the clerkship specific case to the appropriate students: Medicine, Pediatrics, or Neurology.

• Direct them to read their assigned case and discuss the first 3 questions in their handout:

**1.** **Medicine case**: What are the possible reasons for this patient’s recurrent admissions?

***For faculty- possible answers****: Recurrent ischemia, occlusion of bypass graft, non-adherence to medication or medication regimen, dietary indiscretions, environmental causes (housing issues), issues with health information fluency,*

*socioeconomic causes (employment, insurance)*

**Neuro case**: What are the possible reasons for this patient’s admission?

***For faculty- possible answers****: Mechanical fall due to poor ambulatory*

*function Recurrent aspiration pneumonia, Infected sacral wound,*

*Environmental causes due to the layout of the patients home, issues with health information fluency, socioeconomic causes*

**Peds case**: What is the differential diagnosis for this patient’s problem?

***For faculty- possible answers****:*

*The differential for fatigue is enormous and is not limited to: sleep apnea (obese and chronic snoring), inadequate sleep (should get 9-11 hours per night, getting just over 8), poor sleep hygiene (2 others sleeping in room with differing sleep schedules, tablet before bed), anxiety/stress, infection (he has no signs or symptoms), anemia, hypothyroidism*

*The underlying chronic problem is longstanding obesity causing sleep apnea.*

2. Which social determinants of health may be contributing to the patient’s health outcomes?

**Medicine Case:**

***For faculty- possible answers****:*

▪ *Economic: can the patient afford the new medication entresto; are there any economic barriers to the patient medication and medical supplies*

▪ *Environment: What is the patient’s living situation and daily routine*

▪ *Health information fluency: Does the patient understand her medical condition? Does she understand the medications rx’d, expected effects and most common side effects?*

▪ *Food: how are meals prepared, how often does the patient eat; is she following a low Na diet*

▪ *Community and Social Context/Housing Security: is the patient domiciled or at risk for homelessness*

**Neuro Case:**

***For faculty- possible answers****:*

▪ *Economic: Can the patient afford a home health aid given that he and his wife are supporting his son? What other economic barriers exist?*

▪ *Environment: What is the patient's living situation? Does he live in a home or apartment? Are there stairs? Can he manage with his rolling walker?*

▪ *Education: Did the patient receive dietary education regarding a pureed diet during his hospital or rehab stay? Did the patient receive information about how to care for and prevent progression of skin breakdown?*

▪ *Food: Does the patient understand how to prepare a pureed diet? Does the patient have the kitchen tools required to prepare a pureed diet? How*

*often does the patient eat? Is he following the diet recommended during his hospital stay?*

▪ *Community and Social Context: What is the patient's support network?*

**Peds Case:**

***For faculty- possible answers****:*

• *Economic: Can the family afford healthy food? How much does the*

*patient’s mother have to work in order to afford basic necessities such*

*as food/housing/clothes? How does that impact child care options for*

*Bobby and thus his activity level? Can the family afford healthy*

*activities for Bobby outside of school? Do reasonably priced or free*

*activities exist?*

• *Environmental: How safe is Bobby’s neighborhood? Can he play*

*outside in his backyard? Are there nearby parks? What is his living*

*situation and how is it impacting his sleep?*

• *Food: Who is cooking Bobby’s food and what is his access to food?*

• *Health Information Fluency: Does Bobby’s mother understand that*

*he is obese and the potential short and long term impacts on his health? Does she know steps she can take at home to ameliorate it?*

***Section 2: Role Play (45 minutes)***

• Tell students that they will be role playing within their groups of 3 and that there are 3 roles, one for each of them. They can choose amongst themselves which of the following roles to play. Groups with 4 students should have 2 doctors. Groups with 2 students should not have an observer. As you implement this activity, remind students to be actively engaged in role play with the expectation that they will encounter similar scenarios in clinical practice.

o **Doctor**: This student will be asking the patient questions around his/her social determinants of health (determined by the group in Section 1).

o **Patient:** This student will be provided with a detailed social history and will play the patient.

o **Observer:** This student will be provided with a checklist with questions around social determinants and communication skills pertinent to the case. He/she should actively listen for the checklist items during the role play, take notes, and be prepared to provide feedback to the student playing the role of the doctor.

• Set up and run role play (*15 minutes)*

o Instruct the student who will be the doctor to start thinking about how they will frame the questions.

o Instruct student who will be role playing the patient to read the detailed social history.

o Instruct student who will be role playing the observer to read the observer checklist and the social history and to take notes on the checklist during the role play.

o When everyone is ready, the “doctor” should practice asking questions regarding the social determinants the group deemed important to the patient’s problem in Section 1.

The following are possible ways in which the doctor might ask the questions for each case:

**Medicine Case:**

***For faculty- possible answers****:*

• *Are you having any problems filling these medications or supplies?* • *Does anything interfere with your ability to take the medications?* • *What’s your daily routine like?*

• *How frequently are you changing your dressings and how are you doing the dressing changes?*

• *Can you explain to me why you’re taking these medications?*

• *What are the effects of these medications?*

• *What’s your current diet?*

**Neuro Case:**

***For faculty- possible answers****:*

• *Are you having any problems navigating your surroundings?*

• *What is your daily routine?*

• *How has your daily routine been impacted by your new diagnosis?* • *How have you been able to adapt your routine to meet the expectations outlined by your discharge?*

• *Can you explain to me why you are advised to follow a pureed diet?* • *Can you explain to me how you are caring for your sacral wound?*

**Peds Case:**

***For faculty- possible answers****:*

• *What is your living situation? Does Bobby have his own room or does he share a room?*

• *Who is Bobby’s primary caretaker? For example, who watches Bobby and prepares his meals when he is not at school?*

• *Why doesn’t Bobby eat breakfast?*

• *What do you (Bobby’s mother) do during the day?*

• *Are you struggling with affording food, housing, or clothes?*

• *Are you comfortable with Bobby playing outside?*

• *Is there any opportunity for Bobby to get exercise? Are there parks nearby your home?*

• Debrief the role play *(10 minutes)*

o Remind them to start with what was done effectively before moving to opportunities for improvement.

o Start by asking the student(s) who played the doctor to self-assess-What did they do well, what would they have done differently?

o Have the observer debrief the role play in triads using the checklist provided as a guide.

• Replay *(10 minutes)*

o Give the doctor the opportunity to re-practice asking questions and/or to ask missed questions. Suggest they focus on one or 2 social determinants categories.

***Section 3: Identify next steps and early interventions and Prepare case presentation* (*15 minutes)***

• Instruct the students to discuss the next 2 questions in their triads and prepare a short patient presentation for the group:

o Q1: Using the web resources provided, what are your next steps for this patient? For the Medicine and Neuro cases, include a discharge plan.

Medicine case: www.hitesite.org (or use any site specific to your state that provides a resource directory for free and low-cost health and social services)

Patient is concerned about eviction.

Housing Services --. Eviction Prevention. Patient zip code: 11435

Look for services 5- 10 miles from her apartment

Neuro case: www.hitesite.org (or use any site specific to your state that provides a resource directory for free and low-cost health and social services)

Family really needs coordination of care and services

Social Support & Services → Care Coordination Services. Patient zip 11501. Look for 1-5 miles from the ZIP. Need closest center, sort by distance

Pediatric Case: www.hitesite.org (or use any site specific to your state that provides a resource directory for free and low-cost health and social services)

Family needs recreational activities that are safe

Youth & Family Services → After school program. Recreation Programs

Patient zip 11549. Look for 10 miles from the ZIP. Need closest center, sort by distance

o Q2: What steps could have been taken earlier in this patient’s care to prevent the downstream consequences to this patient’s health?

**Medicine Case:**

***For faculty- possible answers****:*

*1. Explain medications and effects*

*2. Check patient’s Health Information Fluency: Discuss the Teach Back Method*

*3. Check insurance and affordability of medications as part of your discharge plan*

*4. Ensure that patient is taught dressing changes correctly and ask how/where will she be able to change the sternal wound dressings*

**Neuro Case:**

***For faculty- possible answers****:*

*1. Consider speech and swallow tests in patients post CVA (medical knowledge) 2. Inquire about living situation before discharge post CVA*

*3. Inquire about caretakers and asses for services prior to discharge post CVA* **Peds Case:**

***For faculty- possible answers****:*

*1. Explore socioeconomic status with patient’s mother*

*2. Assess health information fluency in mother*

*3. Ask if there were any impediments to allowing her child to exercise (money, safety, lack of knowledge of places, no parks)*

• Instruct each triad to prepare a brief (under 7 minutes) patient presentation for the group and designate one person to give it. The presentations should include:

i. A brief medical summary

ii. The social determinants of health that impacted this patient’s clinical

outcomes

iii. Preventative measures that could have been taken to improve this patient’s health

iv. Next steps for this patient

***Section 4: Group Presentations (20 minutes, roughly 7 min per presentation)***

One member from each group:

2. Presents their patient including:

a. Clinical summary of the patient

b. The social determinants of health that impacted this patient’s clinical outcomes c. Preventative measures that could have been taken to improve this patient’s health d. Next steps for this patient

The following are example clinical summaries (2a). Students will be expected to answer all 4 sections above (2b,c,d).

***Medicine Sample Clinical Summary:***

*The patient is a 56 y/o woman with hypertension and hyperlipidemia, who p/w dyspnea and is found to have a myocardial infarction with congestive heart failure (CHF). She was cathed and CABG was recommended. After successfully being diuresed and surgically revascularized, she was discharged with diuretics, CAD medication including a new CHF drug, and dressing changes for her sternal wound. Days later the patient re-presented with decompensated CHF. She wasn’t adherent to the medication as prescribed. She was diuresed and d/c’ with instructions to take medications especially the Furosemide and the new CHF med Sacubitril-Valsartan. Several days later, she again presented and was admitted to the hospital with CHF and now a probable sternal wound infection with dehiscence.*

***Neuro Sample Clinical Summary:***

*The patient is a 77 year old man with diabetes, hypertension, hyperlipidemia and osteoarthritis who presented with right sided weakness and was found to have a Left MCA infarct. His blood pressure treatment was optimized. During his hospital stay he was found to have a right lower lobe pneumonia and a speech and swallow evaluation recommended thickened liquids and a pureed diet. The patient spent two weeks in a rehab facility and developed a stage 1 sacral ulcer. He was discharged with a rolling walker. Upon returning home, the patient struggled to manage his two story home. He had been the primary caregiver for his wife who has severe rheumatoid arthritis. He has done the shopping and food preparation, however following the stroke is not able to drive and has been relying on food brought to the home by neighbors. Additionally the patient has had difficulty following the wound care instructions and has not obtained the supplies recommended. He presents following a fall and is also noted to be febrile with a cough and increased sacral skin breakdown.*

***Peds Sample Clinical Summary:***

*Bobby is an 8 year old boy with a history of reactive airway disease as a toddler and longstanding exogenous obesity presenting with recent fatigue and a drop in school performance. He has been snoring heavily for the past 2 years. He sleeps just over 8 hours per night. His sleep is interrupted nightly by his mother and brother who share his bedroom and keep different sleeping hours. Bobby’s diet is high in fat and sugar. He is unable to eat breakfast because he wakes up late for school due to fatigue. As his mother works long hours, his primary caretaker is his adolescent cousin who is frequently preoccupied. This, combined with a neighborhood his mother deems unsafe, leads him to have 6+ hours of screen time per night with no physical activity outside of school gym class. He likes soccer but does not have the opportunity to play. Review of systems is negative other than snoring. Physical exam is significant for obesity, 2+ tonsils, no goiter.*

***Section 5: Putting it All Together (10 minutes)***

• Pose the following questions to the students:

o We know that time constraints make it impossible to cover all aspects of patients’ social determinants of health. Given all that we have covered today, are there one or two questions that could open the conversation with patients around social determinants and allow you to hone in on those that are most relevant?

• **For faculty, possible answer to the question: Can you name 2 or 3 issues that might be causing you a great deal of stress in your life right now?**

*Background for faculty (this info is in the student guide):*

*Our goal is to improve the total health of our patients. According to research: Overall or total health = 20% Clinical Care +30% Health Behaviors (Diet/Sexual Activity, Exercise) + 40% Socioeconomic factors (Employment, Income, Community Safety) + 10% Physical Environmental Factors (Air quality, Housing)*

***Section 5: Wrap Up (5 minutes)***

• Ask each student to discuss one lesson they learned from this session that they can apply in their next clerkship.
